# Imputation of Ancient Whole Genome *Sus scrofa* DNA Introduces Biases Toward Main Population Components in the Reference Panel

**DOI:** 10.3389/fgene.2022.872486

**Published:** 2022-07-12

**Authors:** J. A. M. Erven, C. Çakirlar, D. G. Bradley, D. C. M. Raemaekers, O. Madsen

**Affiliations:** ^1^ Groningen Institute of Archaeology, University of Groningen, Groningen, Netherlands; ^2^ Smurfit Institute of Genetics, Trinity College Dublin, Dublin, Ireland; ^3^ Animal Breeding and Genomics, Wageningen University and Research, Wageningen, Netherlands

**Keywords:** imputation, ancient DNA (aDNA), *Sus scrofa*, animal husbandry, Neolithic

## Abstract

Sequencing ancient DNA to high coverage is often limited by sample quality and cost. Imputing missing genotypes can potentially increase information content and quality of ancient data, but requires different computational approaches than modern DNA imputation. Ancient imputation beyond humans has not been investigated. In this study we report results of a systematic evaluation of imputation of three whole genome ancient *Sus scrofa* samples from the Early and Late Neolithic (∼7,100–4,500 BP), to test the utility of imputation. We show how issues like genetic architecture and, reference panel divergence, composition and size affect imputation accuracy. We evaluate a variety of imputation methods, including Beagle5, GLIMPSE, and Impute5 with varying filters, pipelines, and variant calling methods. We achieved genotype concordance in most cases reaching above 90%; with the highest being 98% with ∼2,000,000 variants recovered using GLIMPSE. Despite this high concordance the sources of diversity present in the genotypes called in the original high coverage genomes were not equally imputed leading to biases in downstream analyses; a trend toward genotypes most common in the reference panel is observed. This demonstrates that the current reference panel does not possess the full diversity needed for accurate imputation of ancient *Sus*, due to missing variations from Near Eastern and Mesolithic wild boar. Imputation of ancient *Sus scrofa* holds potential but should be approached with caution due to these biases, and suggests that there is no universal approach for imputation of non-human ancient species.

## 1 Introduction

Recent advances in sequencing techniques led to a dramatic increase in the amount of retrievable ancient DNA (aDNA) from archaeological remains (Kircher, 2012), providing new insights into recent evolutionary history (Slatkin and Recimo, 2016; MacHugh et al., 2017; Brunson and Reich, 2019; McHugo et al., 2019). Poor preservation and contamination of exogenous DNA restricts sequence quality, reliability, and coverage of aDNA from archaeological bones (Pääbo et al., 2004; Prüfer et al., 2010). Furthermore, the damaged nature of aDNA poses computational challenges and introduces biases to the analysis of aDNA (Höss et al., 1996; Brotherton et al., 2007; Briggs et al., 2009; Prüfer et al., 2010; Ginolhac et al., 2011; Sánchez-Quinto et al., 2012; Parks and Lambert, 2015; Kistler et al., 2017). One way to counter these problems is imputation, which is a powerful way to improve the quality of data and can potentially maximize the power of analysis that require dense genotypes such as runs of homozygosity (ROH), in depth admixture and trait association analyses (Gamba et al., 2014; Martiniano et al., 2017). Imputation is widely employed in studies of modern data (Van den Berg et al., 2019; Ye, et al., 2019), targeting allele frequencies from a set of reference individuals to infer allele frequencies at unknown or missing sites (Browning and Browning, 2007; Ausmees, 2019).

In aDNA studies, imputation has been applied on human genomes and achieved high levels of concordance between imputed genotypes and their high-quality (HQ) counterparts (>99%) (Gamba et al., 2014; Martiniano et al., 2017; Ausmees et al., 2021). Imputation of aDNA beyond humans is lacking; livestock aDNA is critical to understand pivotal moments in recent evolution such as domestication and pose an excellent case study. A number of factors are known to influence imputation ranging from reference panel characteristics to demographic history; assessing the potential and limitations of imputation of species beyond model species like humans is valuable to aid our understanding of not only imputation performance but also recent evolutionary events.

This paper assesses the power of imputation to increase the quality and information potential of low coverage aDNA samples, using *Sus scrofa* as a case study. This species is an intensively studied livestock species in terms of aDNA, particularly in the context of expansion of animal husbandry into Europe and significantly enhancing our understanding of how farming started in Europe (Larson et al., 2007; Ottoni et al., 2013; Frantz et al., 2019). Investigations have indicated that ancient Near Eastern domestic pigs lost their Near Eastern genomic signatures after their introduction to Europe (Larson et al., 2007; Frantz et al., 2019). Obtaining HQ samples to pinpoint the pace and nature of this turnover in different regions and shorter timescales in relation to larger societal and economic developments is necessary, but it remains a challenge due to poor preservation and contamination. To address this challenge, a systematic evaluation of different imputation methods was performed on whole genome ancient *Sus scrofa* DNA using data from a recent study consisting of ancient whole genomes of pigs sequenced to an appropriate depth for imputation (Frantz et al., 2019). Imputation achieved high genotype concordance but this is paired with biases toward a fraction of the reference panel. These biases might be related to the size and diversity of the reference panel, the reference genome, or the genetic architecture of pigs, and they impose limitations on the interpretive power of imputed data in terms of the proposed genomic turnover of this species in particular and in general the evolution of animal husbandry in Neolithic Europe.

## 2 Materials and Methods

Evaluating imputation of *Sus scrofa* aDNA by comparing three tools, two pipelines, and three variant calling methods.

### 2.1 Data Description and Preparation

#### 2.1.1 Ancient Samples

Seven archaeological samples with high-coverage data and four archaeological samples with moderate coverage from Frantz et al. (2019) were used (Table 1; Supplementary Table S1). Raw FASTQ reads were downloaded from the ENA (accession numbers see Supplementary Table S1). Raw reads were trimmed using *cutadapt v2.10* (Martin, 2011) for quality (<20), length (<20) and adapters used in the library preparation (Meyer and Kircher, 2010). *FastQC v0.11.9* quality reports were made for the raw and trimmed data (Andrews, 2010). The trimmed reads were aligned applying the *Burrows-Wheeler algorithm (BWA) aln v0.7.17* (Li and Durbin, 2009) to the *Sus scrofa* 11.1 reference genome (Warr et al., 2020), with default parameters apart from disabling the seed option (−l 1024), increasing the maximum number of gap opens (−o 2) and changing the maximum edit distance (−n 0.01). Duplicates were removed with *Picard MarkDuplicates v2.18.17* (http://broadinstitute.github.io/picard) and BAM files from different sequencing lanes were merged using *SAMtools merge v0.1.19* (Li et al., 2009). Duplicates were removed with *FilterUniqueSamCons*.py for the merged BAM files (Kircher, 2012). Indels were realigned with *GATK 3.8 RealignerTargetCreator* and *IndelRealigner* with default parameters (Van der Auwera et al., 2013). Depth of coverage and quality were computed using *Qualimap v2.2.1* (Okonechnikov et al., 2015). Molecular damage was assessed using *MapDamage2.0* using default parameters (Jónsson et al., 2013).

**TABLE 1:** Sample information. ID, origin, period and ancestry taken from Frantz et al. (2019).

ID	Origin	Period	Ancestry	Genome coverage
KD033	Germany-Herxheim	Neolithic	∼46% European, ∼54% Near Eastern	6.9
KD037	Germany-Herxheim	Neolithic	∼91% European, ∼9 Near Eastern	21.6
VEM185	England-Durrington Walls	Neolithic/Bronze Transition	∼90% European, ∼10 Near Eastern	21.7

Contamination from prokaryotes and humans was assessed by calculating percent identity score and coverage per read with *BLAST + Blastn Megablast v2.10.1* on prokaryotes, human and *Sus scrofa* databases (Camacho et al., 2008). Reads were considered contaminants if the percent identity (E-value) and coverage of the contaminants (prokaryotes and humans) was higher than the percent identity and coverage of *Sus scrofa*. Contaminated reads were removed from the BAM file with a custom-made python script.

Imputation was assessed by comparing imputed genotypes to their corresponding HQ genotypes, similar to previous studies (Gamba et al., 2014; Martiniano et al., 2017; Ausmees et al., 2021). Three of the seven samples with high-coverage data (KD033, KD037, and VEM185) were downsampled with *Picard v2.18.17* (http://broadinstitute.github.io/picard), to create low coverage samples for imputation ranging from 0.5 to 2× with steps of 0.5×. Three methods were used to assess the accuracy of imputation: Method 1, imputation with variant sites; Method 2, imputation with all confident sites; and Method 3, added to achieve higher genotype concordance which called only genotypes present in the reference panel. HQ genotypes were created from the high-coverage samples to create a golden standard. Genotype likelihoods were called with the *Genome Analysis Toolkit (GATK) UnifiedGenotyper v3.8.0* (Van der Auwera et al., 2013) using either each alignment data of the ancient samples individually or by joined SNP calling. Genotype likelihoods were called with a minimum quality of 25, with output mode *EMIT_VARIANTS_ONLY* for Method 1, *EMIT_ALL_CONFIDENT_SITES* for Method 2 and output mode EMIT_ALL_SITES and genotyping mode GENOTYPE_GIVEN_ALLELES for Method 3, with -alleles genotypes from the reference panel. Variants were filtered to keep only autosomal, biallelic SNPs, and a minimum quality of 30. In order to avoid introducing a possible bias from nucleotide misincorporations due to post-mortem damage, the generated VCF (Variant Call Format) files were filtered to exclude all sites where the most likely genotype could have been inferred from a deaminated allele with a custom-made python script. For C→T deaminations, C**↔**T SNPs were excluded from further analyses if the most likely genotype contained a T allele, and for G→A deaminations, G**↔**A SNPs were excluded from further analyses if the most likely genotype contained an A allele. Genotypes were not filtered in Method 3 when using GLIMPSE, because this software only imputes genotypes present in the target VCF, they were instead kept as no calls (./.).

#### 2.1.2 Reference Panel

The reference material used for imputation consisted of the wild boar and pig breeds collection of Wageningen University and two Iberian samples from Ramírez et al. (2015) (Supplementary Table S1). Pig breeds that have no known introgression with Asian breeds were selected to avoid potential bias. Genotype likelihoods were called with the *Genome Analysis Toolkit (GATK) UnifiedGenotyper v3.8.0* (Van der Auwera et al., 2013), with a minimum quality of 15, calling SNPs, with the mode *EMIT_VARIANTS_ONLY* for Method 1 and *EMIT_ALL_CONFIDENT_SITES* for Method 2. The reference panel was filtered to only include autosomal biallelic SNPs, a minimum quality of 30, and a minimum depth of 4, a call rate of 0.8, and removal of repetitive elements. In order to evaluate the effect of the reference composition on the imputation, multiple reference panels were considered. The main reference panel consists of modern pig breeds, European wild boar, and Near Eastern wild boar (51 individuals, 12,737,362 variants—Table 2; Supplementary Table S2). To deduce the effect of ancient samples on imputation, eight ancient individuals were added to the main reference panel, consisting of two Near Eastern samples, three ancient Near Eastern, and three ancient European samples (59 individuals, 10,823,257 variants—Table 2, Supplementary Table S2), called Main + ancient reference. Moreover, the main reference panel was divided into several subsets to pinpoint the effect of reference bias on imputation (See Supplementary Material-Subsets of reference panel). Additionally, to deduce the effect of Asian haplotypes on imputation, Asian wild boars, Asian domestic pigs and South-East Asian *Sus* were added to the reference panel (See Supplementary Material-Including Asian samples). Different filters and combinations of filters were used on the reference panel to optimize the imputation workflow and deduce the effects of these filters on imputation. These filters consisted of removing 1) transversions, 2) transitions, 3) filtering for minor allele frequency (MAF) bins {<0.05, 0.05–0.1, 0.1–0.3, >0.05, >0.3, No MAF}, and their various combinations. Results of all combinations can be found in Supplementary Table S2. The reference panels were phased with *Beagle5* (Browning et al., 2018), using default parameters apart from changing the effective population size (Ne) to 20,000 (Groenen et al., 2012).

**TABLE 2:** Reference panels with their respective number of individuals/population.

Reference panel	Number of individuals
Main references	
Dutch wild boar-European wild boar (EUW)	12
Italian wild boar-European wild boar (EUW)	6
French wild boar-European wild boar (EUW)	1
Pig breeds-European Domestic (EUD)	25
Greek wild boar (BLW)	4
Near Eastern + Turkish wild boar- Near Eastern wild boar (NEW)	3
Total	51
Main + ancient references	
Main reference	51
Near Eastern-Ancients (ANC)	5
European-Ancients (ANC)	3
Total	59

### 2.2 Genetic Map

A genetic map was created using the recombination frequencies that Johnsson et al. (2020) estimated based on nine genotyped pedigrees on the *Sus scrofa* 11.1 reference genome. These recombination frequencies were converted to cM using the Haldane formula (Haldane’s Mapping Function, 2008). Genetic maps were made for each chromosome in the plink format with bins of 1 MB (Supplementary Table S3).

### 2.3 Imputation

For Methods 1–3 imputation was performed using *Impute5* and *Beagle5*, using default parameters, with a phased reference panel (Supplementary Table S2), with a *Ne* of 20,000 and, --div-select and –out-gp-field parameters for *Impute5* (Rubinacci et al., 2020) and window = 40, overlap = 4 and gp = true parameters for *Beagle5* (Browning et al., 2018). Imputation was performed for chromosome 1–18, individually and using sliding windows (See Supplementary Material—Chromosomal imputation). The effect of including multiple ancient samples on imputation was evaluated by imputing joint ancient samples and was compared to individual imputation (Supplementary Material—Joined Imputation). The focus was on individual imputation. Imputation was performed using two different imputation pipelines: 1) the original one-step pipeline used in Ausmees et al. (2021) and 2) the two-step pipeline used for low coverage samples in Hui et al. (2020). The two-step pipeline adds another filtering step prior to imputation that accounts for genotype probability. *Beagle 4.1* was used to calculate genotype probabilities for the target downsampled VCF using default parameters, with the same phased reference panel that was used for imputation (Supplementary Table S2), with a Ne of 20,000 and gprobs = true parameters. Variants with a genotype probability (GP) < 0.99 were removed from the target downsampled VCF, leaving only confident genotype calls. The imputed genotypes were filtered for an imputation score of 1 (highest imputation accuracy). For Method 3, *GLIMPSE v1.1.1* (Rubinacci, et al., 2021) was also used with similar settings as applied in ancient human imputation and the pipeline proposed by Rubinacci, et al. (2021), with default parameters, and a phased reference panel (Supplementary Table S2). Variants with an imputation score of <1 were removed from the target downsampled VCF, leaving only confident imputed genotype calls. GLIMPSE *v1.1.1* was only tested with Method 3 because of the incompatibility with the other two methods/pipelines.

### 2.4 Genotype Concordance

Imputation accuracy was assessed by genotype concordance defined as the fraction of genotypes that were imputed correctly. This was measured by dividing the incorrectly imputed SNPs with all imputed SNPs and was measured separately for each sample. The correctly and incorrectly imputed SNPs were derived from comparing the imputed SNPs to their HQ counterpart similar to the approach of *Picard GenotypeConcordance*. The HQ genotypes used are pre-deamination filtered, to keep confident transitions (transitions that also occurred in the reference panel), and not transitions arising from deamination. The incorrectly imputed SNPs were classified into incorrect positions (positions not occurring in HQ) and incorrect genotypes (genotypes different from HQ genotypes). Information content, that is, the amount of gained genotypes, was calculated by dividing the amount of imputed genotypes to the total amount of HQ genotypes.

### 2.5 Downstream Analysis

Downstream analyses were performed to investigate the difference and/or similarity between imputed and HQ genotypes. Data were pruned with *PLINK 1.9* (Purcell et al., 2007) with the parameters—geno 0.10. A principal component analysis (PCA) was performed on diploid genotypes consisting of the reference panel, the HQ samples and the imputed samples using *PLINK 1.9* pca on autosomes only. Eigenvalues and vectors were plotted with the use of *Mathplotlib* (Hunter, 2007) and *Seaborn* (https://zenodo.org/record/883859#.XSdFFugza01). An admixture analysis was performed using the same dataset as the PCA analysis, however separately for downsampled, imputed and HQ genotypes. A*DMIXTURE v1.3.0* (David et al., 2009) was used with standard parameters and K ranging from 2 till 5. Furthermore, bootstrapping was performed using the parameter -B. Identical By Descent (IBD) analysis was conducted on the same dataset as the admixture analysis*. IBDseq v2.0* with standard parameters was used to calculate IBD segments between samples (Browning and Browning, 2013). A regions of homozygosity analysis was performed using the same dataset as the admixture analysis using plink –homozyg with the parameters –homozyg-kb 10, --homozyg-gap 10, --homozyg-snp 100, --homozyg-window-het 2, --homozyg-window-snp 100 --homozyg-window-missing 1. *DetectRUNS* (https://cran.r-project.org/package=detectRUNS) was used to visualize and calculate ROH statistics.

### 2.6 Reference Affinity

Correct and incorrect imputed genotypes were compared to their HQ counterpart to assess whether imputed genotypes show a systematic bias toward the reference genome. Reference bias was measured as the presence/absence of different ancestral/origin groups between the correct and incorrect imputed genotypes and their HQ counterpart.

## 3 Results

Genotype concordance was calculated for three imputation tools, two pipelines and three variant calling methods to test the best method to approach imputation in *Sus scrofa*. Downstream analyses were performed to assess the accuracy and power of imputation.

### 3.1 Genotype Concordance

#### 3.1.1 Tools: Beagle5 Versus Impute5

**FIGURE 1:**
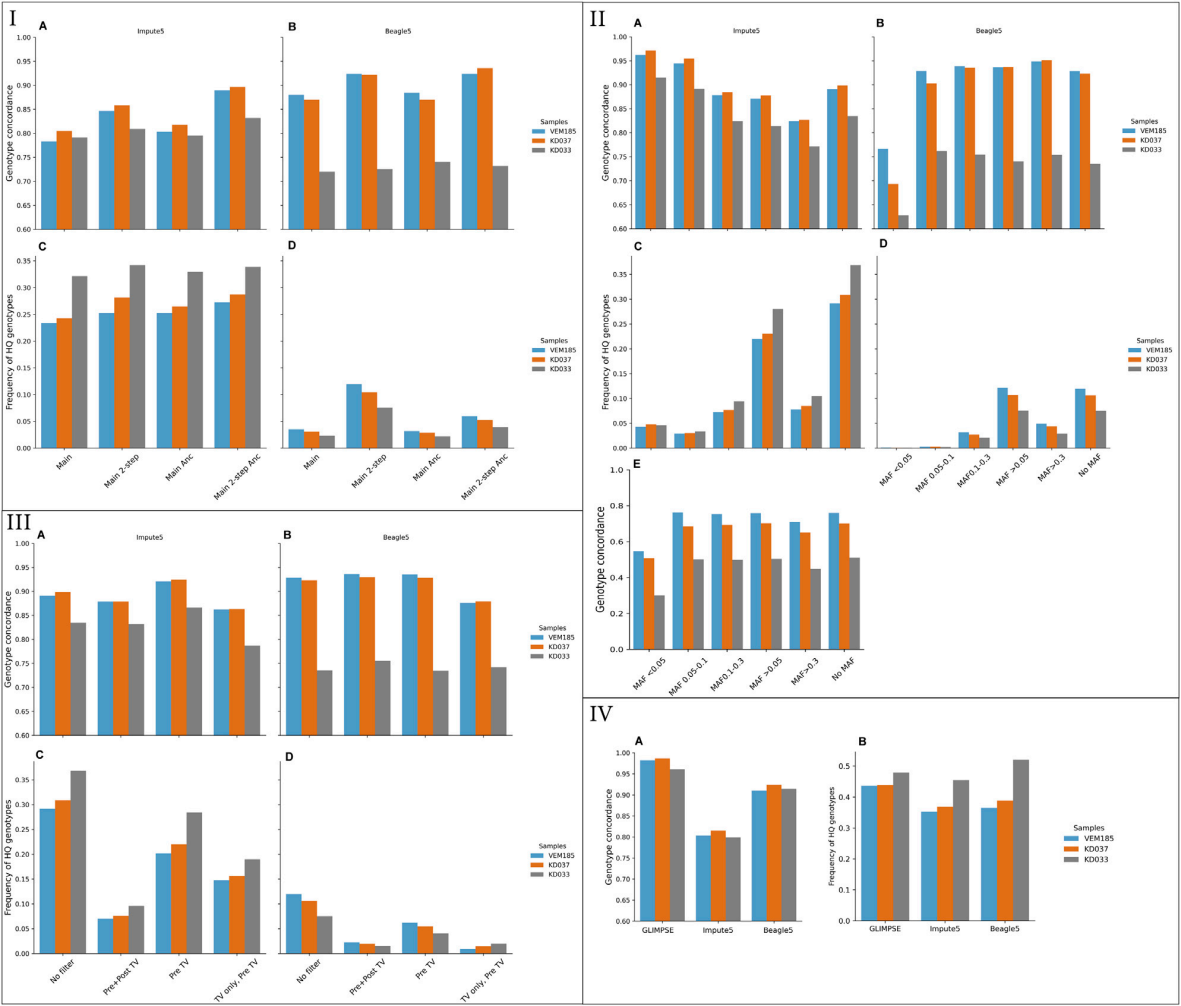
Genotype concordance and fraction of high-quality (HQ) genotypes (portrayed in decimals, total HQ genotypes: VEM185: 4,701,683, KD037: 4,531,126, KD033: 3,887,848) using different imputation tools and reference panels. Imputed from 1× downsampled coverage genomes of VEM185, KD037, and KD033. I) Genotype concordance for Impute 5 **(A)**, Beagle5 **(B)**, Fractions of HQ genotypes covered by imputation in Impute5 **(C)**, and Beagle5 **(D)**. II) Genotype concordance and Fraction of HQ genotypes of various MAF bins of Main + 2-step + Anc + all reference panel. Genotype concordance for Impute 5 **(A)**, Beagle5, **(B)** Fractions of HQ genotypes covered by imputation in Impute5 **(C)**, Beagle5 **(D)** and, Genotype concordance of only heterozygotic variants of various MAF bins imputed with Impute5 **(E)**. III) Filters on target downsampled vcf. Genotype concordance for Impute 5 **(A)**, Beagle5 **(B)**, Fractions of HQ genotypes covered by imputation in Impute5, **(C)** Beagle5 **(D)**. IV) Genotype concordance **(A)** and fraction of HQ genotypes **(B)** from GLIMPSE, Impute5 and Beagle5 for Method 3. * Genotype concordance of Method 3 was done on all sites and cannot be directly compared to the other methods. Main, original one-step pipeline on main reference panel; 2-step, two-step pipeline with an extra filtering step; Anc, Ancient samples included in the reference panel; TV, transversions.

Genotype concordance was higher for Beagle5 compared to Impute5 for KD037 and VEM185 but lower for KD033 (Figure 1). For both tools, KD037 and VEM185 performed better than KD033, this being more pronounced for Beagle5. The amount of correctly imputed variants differed greatly between the tools, with Beagle5 being systematically lower (Figures 1IC,D). Impute5 imputed 25%–34% of the total amount of HQ genotypes, while Beagle5 imputed around 5%. Beagle5 achieved the highest genotype concordances in KD037 and VEM185, but produced less correctly imputed variants. Impute5, on the other hand, achieved the highest genotype concordance in KD033 and produced more correctly imputed variants. Furthermore, genotype concordance between chromosomes was more uniform in Beagle5 compared to Impute5 (Supplementary Table S4). These results are based on the default one-step pipeline. The main one-step pipeline was extended to the two-step pipeline to test various settings for both tools that could influence imputation accuracy changing one element at a time.

#### 3.1.2 Pipeline: One-Step Versus Two-Step

Genotype concordance in the two-step pipeline was higher compared to the one-step pipeline for both tools (Figures 1IA,B). The amount of correctly imputed variants increased for Beagle5 whereas the amount of correctly imputed variants for Impute5 stagnated (Figures 1IC,D). Genotype concordance between chromosomes was more uniform in the two-step pipeline compared to the one-step pipeline (Supplementary Table S4) (variation of filter combinations used for the comparisons can be found in Supplementary Table S2).

#### 3.1.3 Reference Panel: With and Without Ancient Samples

Genotype concordance was ∼5% higher in the two-step pipeline when using the reference panel including ancient samples for Impute5, whereas the inclusion of ancient samples only provided a 0.5% different genotype concordance for Beagle5 (Figures 1IA,B; Supplementary Table S4). Similarly, genotype concordance between chromosomes showed more uniformity with ancient samples than without ancient samples for Impute5, but not for Beagle5 (Supplementary Table S4). The amount of correctly imputed variants with respect to the inclusion of ancient samples had no effect for Impute5 but decreased for Beagle5 (Figures 1IC,D).

#### 3.1.4 Reference Panel: Variant Sites Versus All Confident Sites Category (All)

Using the *all confident sites* category, method 2, slightly increased genotype concordance for both tools (Supplementary Figures S1A,B). The amount of correctly imputed variants was larger in the *all confident sites* category compared to the *variant sites* category, method 1, for both tools (Supplementary Figures S1C,D). The uniformity of genotype concordance between chromosomes was more equal in the all confident sites category compared to the variant sites category for Beagle5, except for KD037 (Supplementary Table S4).

#### 3.1.5 Reference Panel: Pre-Imputation Filters Versus Standard

Reference panels filtered for transversions and transitions showed similar genotype concordance, with transversions only having the lowest genotype concordance (Supplementary Figures 2A,B). The amount of correctly imputed variants decreased drastically for only transitions and only transversions with roughly 50%, in both tools (Supplementary Figures 2C,D). The uniformity of concordance between chromosomes was equal for all filters (Supplementary Table S4).

**FIGURE 2:**
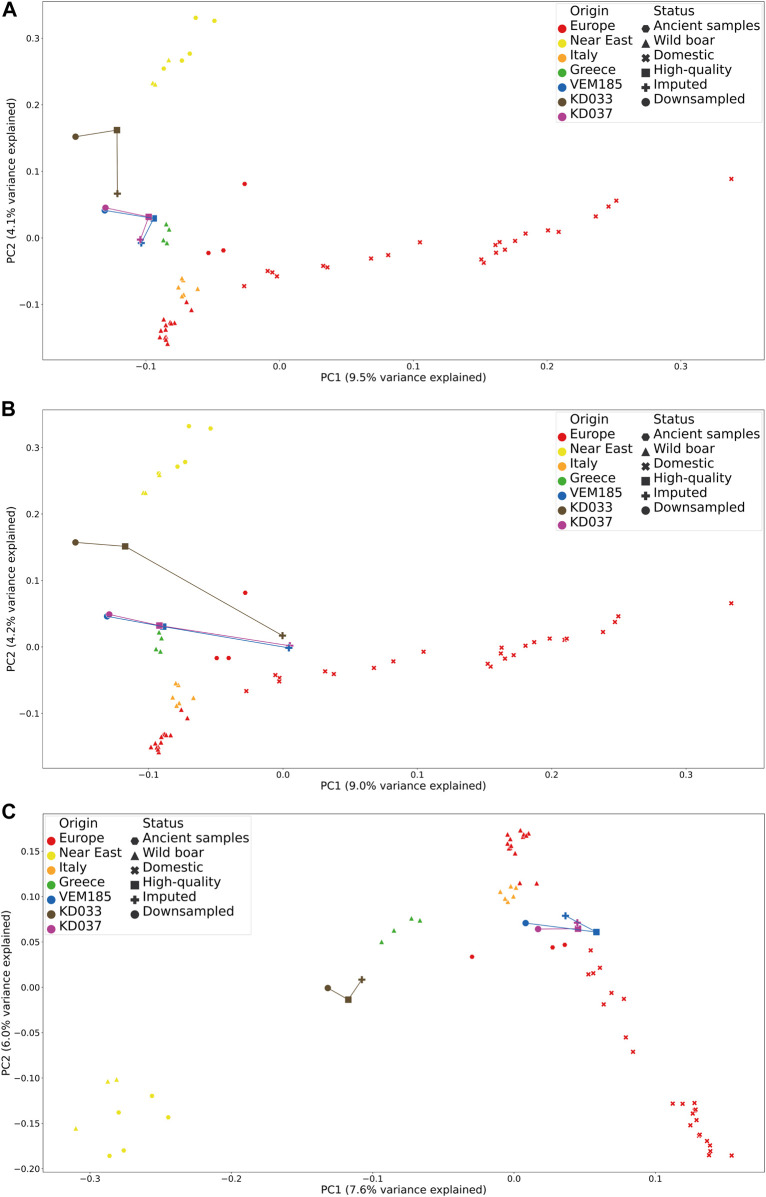
Principal component analysis comparing high-quality, imputed genotypes and downsampled data together with samples from the reference panel. IMP1 **(A)**, IMP4 **(B)**, and IMP5 **(C)**.

Reference panels filtered for MAF showed variation in genotype concordance, where MAF bins <0.05 and 0.05–0.10 had the highest and MAF >0.3 the lowest genotype concordance for Impute5 (Figure 1IIA). This contrasted with Beagle5, where MAF <0.05 had the lowest genotype concordance and MAF >0.3 the highest (Figure 2B). Filtering for MAF (Beside the common variant >0.05 filter) drastically decreased the amount of correctly imputed variants in both tools (Figures 1IIC,D). Genotype concordance of heterozygotes did not show the same trend as all variant genotype concordance for Impute5 (Figure 1IIE), ∼30% of the total imputed genotypes were heterozygotes (Supplementary Table S4). Genotype concordance of MAF bin <0.05 decreased drastically, while the other MAF bins decreased more modestly.

#### 3.1.6 Target VCF: Filters Versus No Filters

The target VCF was filtered pre- and post-imputation to deduce the effect on genotype concordance. The reference panels used for this analysis were IMP1 and IMP2. Filtering for transitions pre-imputation, thus only keeping transversions, had the most favorable effect on genotype concordance. The highest genotype concordance for Beagle5 was filtering for transitions pre- and post-imputation, whereas the highest genotype concordance for Impute5 was filtering for transitions pre-imputation (Figures 1IIIA,B). The amount of correctly imputed variants of the pre- and post-imputation filters decreased drastically compared to using no filter on the target VCF for both tools (Figures 1IIIC,D). The imputed variants were further filtered for well-known positions, namely the 50 k porcine SNP-Chip (Yang et al., 2017), the transversions only, and main SNP-sets from the study of Frantz et al. (2019). These filters did not improve genotype concordance and decreased the amount of correctly imputed variants (Supplementary Figure S3 — Methods Known genomic positions).

#### 3.1.7 Target VCF: All Sites (Sites Present in Reference Panel)

A method that has been shown to achieve high genotype concordance in ancient human imputation (Martiniano et al., 2017; Hui, et al., 2020; Method 3) was also applied. This method was tested on three imputation tools, Beagle5, Impute5, and GLIMPSE. GLIMPSE achieved the highest genotype concordance, reaching 98% in KD037 and VEM185 and 96% in KD033 (Figure 1IVA). Genotype concordance for Beagle5 stayed constant for VEM185 and KD037 but increased for KD033 compared to the two-step pipeline. Genotype concordance for Impute5 decreased for all samples compared to the two-step pipeline. The amount of correctly imputed variants increased for all samples and all tools, reaching roughly 50% (Figure 1IVB). Furthermore, the genotype concordance between chromosomes was more constant for this method compared to the two-step pipeline (Supplementary Table S4).

### 3.2 Downstream Analysis

Downstream analyses were performed on the imputed genotypes IMP1, 2, 3, 4, and 5 (Full descriptions found in Table 3). PCA were performed to pinpoint and compare the genetic affinities of imputed, HQ and downsampled samples. Variation captured by the first two principal components of IMP1 shows that the imputed genotypes of KD033, KD037, and VEM185 cluster closer to their HQ counterparts in the first principal component (PC1, 9.5% variation) but tend to have a bias toward European wild boar in the second principal component (PC2, 4.1% variation) (Figure 2A). This bias is greater for KD033 compared to KD037 and VEM185. PCA of IMP2 shows that the downsampled genotypes and the HQ genotypes cluster closer to each other than to the imputed genotypes, the latter showing a bias toward the domestic pigs in the first principal component (PC1, 9% variation) (Supplementary Figure S4). PCA of IMP3 shows that rare alleles have a bias toward the domestic cluster in the first principal component (PC1, 9% variation) and a domestic and European wild boar bias in the second principal component (PC2, 3.8% variation) (Supplementary Figure S5). The PCA for IMP4 has the same trend as IMP2, where the downsampled genotypes and the HQ genotypes cluster closer to each other than to the imputed genotypes. However, the imputed genotypes of KD037 and VEM185 show a decreased bias toward the domestic pigs on the second principal component (PC2, 4.2% variation) (Figure 2B). The PCA for IMP5 shows a bias toward the European wild boar cluster for KD033 and VEM185 in both principal components, where the imputed genotypes of VEM185 cluster closer with the HQ genotypes than the downsampled genotypes (Figure 2C). This trend is not observed in KD033. The imputed genotypes of KD037 cluster closely toward the HQ counterpart, showing a slight bias on PC2 toward the European wild boar cluster. However, there seems to be a slight bias introduced in the HQ and downsampled genotypes, which is more evident for KD037 and VEM185. They are clustering more toward the European domestic cluster than in their previous PCA (IMP1–4). Beagle5 showed a similar trend as GLIMPSE but with an elevated bias toward the downsampled genotypes (Supplementary Figure 6SA). Impute5 showed an increased amount of bias toward the European wild boar cluster (Supplementary Figure 6SB).

**TABLE 3:** Reference panel abbreviations.

ID	Reference panel
IMP1	Main + 2-step + Ancients + All confident sites, Impute5
IMP2	Main + 2-step + Ancients + All confident sites, Beagle5
IMP3	Main + 2-step + Ancients + All confident sites + MAF<0.05, Impute5
IMP4	Main + 2-step + Ancients + All confident sites + MAF>0.3, Beagle5
IMP5	Main + 2-step + Ancients + All, GLIMPSE

**FIGURE 3:**
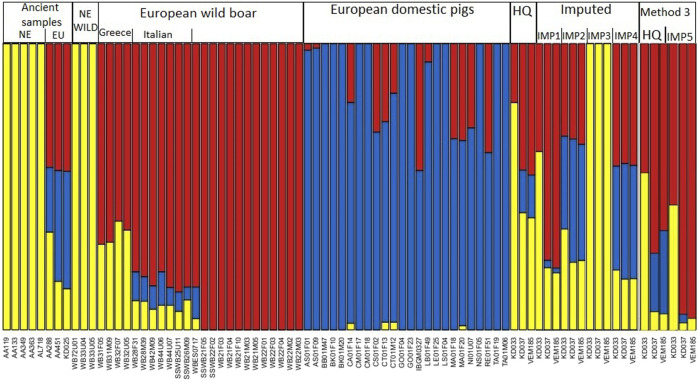
Admixture analysis with K = 3, comparing high-quality and imputed data from IMP1-5 together with samples from the reference panel. NE, Near East; EU, European. The colors represent the three ancestral groups, where blue is European domestic pig, red is European wild boar, and yellow is Near Eastern.

Admixture analysis of three ancestral groups (K = 3) shows the genetic ancestry of reference panel and HQ samples and indicates a presence of all three ancestral groups in KD037 and VEM185, and two ancestral groups in KD033. The ancestral groups of KD037 and VEM185 are similar, with VEM185 having a slightly larger “European domestic pig” component, whereas KD033 consists of a larger Near Eastern and smaller European component. Admixture analysis of IMP1 shows an increase in the European component, and a decrease of the other components of all imputed samples, highlighting a bias toward European samples. Admixture analysis of IMP2 shows a decrease of the European component in KD037 and VEM185 and an increase in KD033. The most noticeable difference between IMP2 and HQ is the increased component of “European domestic pigs” in all three imputed samples, again highlighting a potential bias toward “European domestic pigs.” KD033 showed most bias losing almost half of its Near Eastern component and increasement of both European wild boar and domestic pig’s components. Admixture analysis of IMP3 shows only one ancestral component, namely, the Near Eastern component (Figure 3). This potential bias toward the Near Eastern component could have arisen from a low amount of variants present in the imputed genotypes. Admixture analysis of IMP4 is similar to IMP2, with a slight decrease in Near Eastern ancestral component and an increase in European ancestral component, showing a bias toward the “European domestic pigs” component in all samples and a bias toward the European wild boar component in KD033. Admixture analysis for Method 3 shows a deviation between the two HQ, where the HQ in Method 3 shows a decrease in Near Eastern components and an increase in European wild and domestic components. Therefore, IMP5 was compared to the HQ of Method 3. Admixture analysis of IMP5 shows an increase in the European wild boar component in all samples and a decrease of “European domestic pig” and Near Eastern component in KD037 and VEM185, whereas KD033 only has a decrease in the Near Eastern component.

Identity-By-Descent (IBD) analysis shows that the imputed genotypes of IMP1 share large IBD segments with each other, covering whole chromosomes. These IBD segments are not present in their HQ or downsampled counterparts. IMP2 showed more variation with the imputed genotypes resulting in more fragmented IBD segments when compared to IMP1. However, most of the IBD segments do not overlap with the HQ IBD segments. IMP3 and 4 did not have enough depth to perform a proper IBD analysis. ROH analysis shows a similar but less drastic trend. The amount of ROHs was smaller in the imputed samples but they consisted of longer stretches (Table 4). The imputed samples had considerably larger ROHs, some larger than 1 MB, while the HQ samples had smaller fragmented ROHs. The elongated ROH stretches in the imputed samples attributed to a higher Froh compared to the HQ samples (Table 4). However, the ROHs in the imputed samples overlap with the HQ samples but consists of longer stretches (Supplementary Appendix ROH). The ROH analysis was only performed for IMP1, because Beagle5 had a low amount of variants.

**TABLE 4:** ROH statistics of the IMP1 reference panel for all autosomes shown per class of 0–0.5 mb, 0.5–1 mb, and >1 mb.

	0–0.5			0.5–1			>1		
	Count	Sum kb	Froh	Count	Sum kb	Froh	Count	Sum kb	Froh
KD033_HQ	4,496	232,157	0.1025	1	516	0.0002	NA	NA	NA
KD033_Imp1	3,287	343,670	0.1517	17	13,152	0.0058	2	2,377	0.0010
KD037_HQ	4,434	193,928	0.0856	NA	NA	NA	NA	NA	NA
KD037_Imp1	2,743	285,325	0.1259	12	8,626	0.0038	1	1404	0.0006
VEM185_HQ	3,071	124,173	0.0548	NA	NA	NA	NA	NA	NA
VEM185_Imp1	2,585	266,071	0.1174	14	9,909	0.0044	1	1600	0.0007

Each class has three statistics, ROH count, total sum of kb and Froh.

### 3.3 Effects of Coverage on Imputation

**FIGURE 4:**
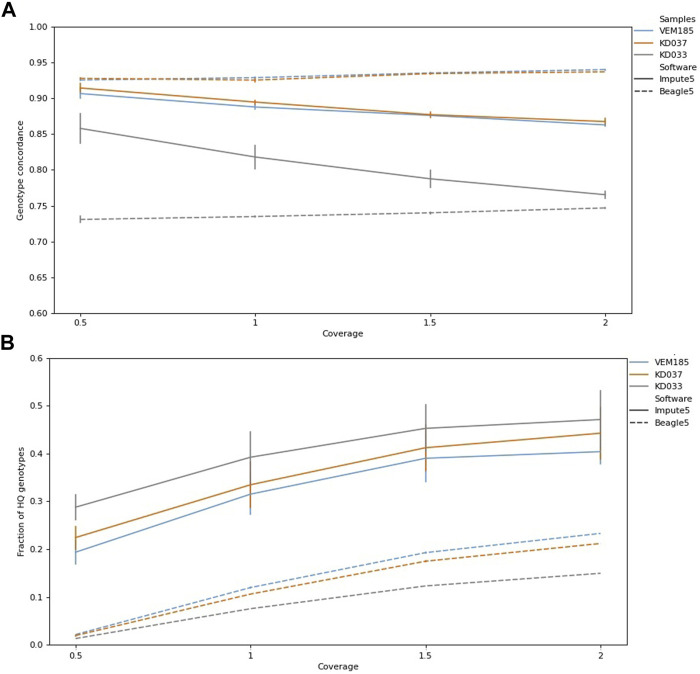
Genotype concordance and fraction of high-quality (HQ) genotypes (total HQ genotypes: VEM185:4,701,683, KD037: 4,531,126, KD033, 3,887,848) of imputed samples for different levels of coverage for Impute5 and Beagle5. Imputation was performed using IMP1 and IMP2. Bars are standard errors. Genotype concordance **(A)**, Fraction of HQ genotypes **(B)**.

Coverage levels vary in genotype concordance, reaching 0.94 for KD037 and VEM185, and 0.75 for KD033 using Beagle5, where 2× reached the highest genotype concordance (Figure 4A). This trend is opposite for Impute5, which reached a genotype concordance of 0.92 for KD037, 0.91 for VEM185, and 0.88 for KD033, with the lowest coverage 0.5×. Imputed genotypes increased with increasing coverage (Figure 4B)

### 3.4 Effects of Reference Bias

The HQ genotypes were considered a baseline of the true genotypes that overlap with the groups in the reference panel (Supplementary Figure S7). For Impute5, the correctly imputed genotypes showed a bias toward the genotype that is most common in the reference panel and occurs across all groups (Ancients, EUW, EUD, and NEW), while the incorrectly imputed genotypes showed a bias toward the EUD; EUW and EUW groups (Supplementary Figures S8,9). For Beagle5, the correctly imputed genotypes showed a bias toward ANC; EUD; EUW, EUD; EUW, EUD; EUW; NEW, and, ANC; EUD, which is similar to the bias shown in the incorrectly imputed genotypes (Supplementary Figures S10,11). The incorrectly imputed genotypes were randomly divided throughout the chromosomes (Supplementary Figure S12).

## 4 Discussion

The analyses revealed that for imputation of *Sus scrofa* aDNA data: 1) genotype concordance is relatively high, similar to modern imputation, with a minor increase in information content (fraction of gained genotypes in relation to HQ) in imputed genotypes and 2) imputation performance showed inaccuracies in downstream analyses. These results have a variety of implications for our understanding of the potency of imputation of non-human ancient DNA in terms of its performance and limitations.

### 4.1 Imputation Performance

The relatively high genotype concordance of 0.95 for Beagle5, 0.925 for Impute5, and 0.98 for GLIMPSE at 1× coverage on ancients is along the lines of genotype concordance in imputation of modern breeds (see Song et al., 2019; Ye et al., 2019; Wang et al., 2021). The higher genotype concordance in KD037 and VEM185 compared to KD033 might be explained by their difference in ancestry. Frantz et al. (2019) have shown that KD033 possessed ∼54% Near Eastern ancestry, while KD037 and VEM185 possessed only ∼10% Near Eastern ancestry (Frantz et al., 2019). The larger component of Near Eastern ancestry in KD033 may have caused the lower performance due to the reference panel being skewed toward European individuals. Another explanation could be the difference in coverage between the samples, KD037 and VEM185 both have coverages >20×, whereas KD033 has a coverage of ∼7x. However, KD037 and VEM185 had the same genotype concordance when downsampled to a similar coverage, excluding this possibility (Supplementary Methods- Downsampling KD037 and VEM185). Moreover, KD033 showed most deviation when downsampled multiple times, showing that the ancestry components of this sample seem to be a factor in the level of accuracy in imputation.

All tools achieved high genotype concordance but differed in amount of information gained. Moreover, Beagle5 showed less variation in imputation of repeated downsampled VCFs compared to Impute5, showing that Beagle5 might be less affected by the randomness of downsampling. Genotype concordance increased with the two-step imputation pipeline. This was specifically designed for genomes with low coverage (Hui et al., 2020). Our results indicate that non-model species and species without an extensive reference panel could also benefit from this approach. Furthermore, genotype concordance increased when ancient samples were added to the reference panel, adding to the number and diversity of individuals. Finally, genotype concordance and number of correctly imputed genotypes increased when using all confident sites but this increased computational time and memory significantly. GLIMPSE achieved the highest genotype concordance with Method 3, that consisted of reference panel called genotypes in the three target downsampled samples. However, this method did not improve the genotype concordance for Impute5 and Beagle5, but did improve amount of genotypes recovered in all tools.

When only looking at genotype concordance the imputation performance of imputation of *Sus scrofa* aDNA could be deemed sufficient. However, there are potential shortcomings. High genotype concordance obtained in the imputed genotypes does not result in an equal representation of genotypes from the original high coverage genome and consists of only a subset, covering roughly 5%–50%. Moreover, imputed genotypes showed greater affinity with populations that are overrepresented in the reference panel as seen in downstream analyses (e.g., PCA, Admixture). One example of the unequal representation of genotypes from the original HQ genome is apparent from genotype concordances on different MAF bins. Genotype concordance in rare alleles (MAF < 0.05) reached 97% but resulted in a bias toward main components in the reference panel in downstream analyses. This is potentially due to the representation of only ∼5% of the original HQ genotypes in the imputed genotypes It is therefore essential to look beyond genotype concordance and focus on multiple aspects like fraction of HQ genotypes obtained by imputation and potential biases in downstream analysis.

Downstream analyses can identify how imputed genotypes act in comparison to their HQ counterpart. The PCA of IMP1 resulted in accurate clustering of imputed and HQ genotypes with only a slight bias toward the European wild boar component. This same analysis for Beagle5 resulted in a stronger bias toward the European domestic pig component. This illustrated that a high genotype concordance does not necessary lead to accurate downstream analyses. The imputed genotypes are correct, but introduce bias in subsequent downstream analyses because they are from specific regions of the genome and are not informative enough to detect genetic variation among samples. This trend is also apparent in the admixture analysis, where imputed genotypes have biases toward European wild boar and domestic pig components. IMP3 is an exception that might be attributed to the high amount of missing genotypes pulling it toward the Near Eastern component that featured missingness, due to low coverage and ancient individuals. IMP5 achieved the highest genotype concordance and resulted in the most accurate clustering for KD037 in the PCA, suggesting that imputation of ancient *Sus* is feasible. However, VEM185 had a similar genotype concordance as KD037 but showed the most bias in downstream analyses for this specific method, implying that high genotype concordance does not preclude bias across samples.

The IBD analysis shows that imputed genotypes of different samples from Impute5, share large IBD segments, sometimes even stretching chromosome wide. This could be a result of: 1) samples which lost their individual variation and became more similar due to imputation and/or 2) imputed genotypes that did not have enough depth for IBD analysis. The second explanation is unlikely, as imputed genotypes for Beagle5, did not show these large IBD segments. The ROH analysis shows that there are longer homozygous stretches throughout the genome in imputed genotypes compared to their HQ counterparts. Causes for this could be that the imputed genotypes were predominantly homozygous with little representation of heterozygotes, contributing to long ROHs and that the imputed genotypes have less markers than the HQ counterparts, resulting in an unequal density of markers. Thus, interpretation of ROHs in imputed ancient *Sus* should be taken with caution as it can be a result of the increase in homozygosity for Impute5. Overall, these downstream analyses highlight that there are biases and limitations toward imputation of *Sus scrofa* aDNA.

### 4.2 Factors Limiting the Power of Imputation of *Sus scrofa* aDNA

One of the limitations is size of the reference panel (59 individuals), but more specifically diversity in the reference panel. Studies on both humans and pigs showed that a larger and more diverse reference panel increase imputation accuracy (Jostins et al., 2011; Pistis et al., 2015; Van Den Berg et al., 2019; Ausmees et al., 2021; Wang et al., 2021). Ancient human imputation studies had a minimum of ∼250 individuals to perform successful imputation (Ausmees, 2019). Adding individuals to the reference panel, that do not add genetic diversity to target samples does not increase genotype concordance, as seen from the results when adding Asian samples to the reference panel. The current reference panel lacks diversity, as the main groups in the reference panel consisted of European wild boar, European domestic pigs and Near Eastern wild boar, with (Dutch) European wild boar and European domestic pig dominating. A study on ancient human imputation observed a lower genotype concordance and similarity in their PCA for hunter-gatherer genomes of which ancestry is more or less absent in the reference panel (Ausmees et al., 2021). This was also found in imputation of pig breeds where rarer pig breeds had a lower genotype concordance and dosage score than breeds that were common in the reference panel (Wang et al., 2021). In this study genotype concordance improved when adding five ancient samples with Near Eastern ancestry to the reference panel. Improving and mitigating current biases of the reference panel should aid imputation. This could be achieved by including Mesolithic wild boar, Iberian, British, Scandinavian and East European wild boar, Near Eastern wild boar and domestic pigs to the reference panel.

Another potential limitation that is associated with the reference panel is the available reference genome. The *Sus scrofa* 11.1 reference genome, is from a Duroc individual, with known Asian introgression. Moreover, the nature of the reference genome could potentially increase the rate of false genotyping leading to errors in haplotypes and LD structure, which could result in decreasing imputation accuracy.

One final potential limitation is the genetic architecture of pigs. Accuracy of imputation is dependent on LD, recombination, genetic distance, and MAF (Stephens and Scheet, 2005; Browning et al., 2018; Ye et al., 2019). These factors are different in pigs compared to humans and even other livestock species, where average heterozygosity is lower and, LD and genetic distance are significantly greater (Zhang and Plastow, 2011). The recombination rate used in this study was based on nine breeding lines, all having introgression with Asian domestic pigs. This potentially introduced inaccuracies but is mitigated as the recombination was divided into bins of 1 MB, which might not be at a size resolution to introduce inaccuracies between wild and domestic pigs.

## 5 Conclusion

The use of imputation of ancient low-coverage *Sus scrofa* data resulted in relatively high genotype concordance and a moderate increase in information content. However, the imputed genotypes represented only a fraction, roughly 5%–50%, of all genotypes called in the HQ ancient genomes and featured biases toward the main population components in the reference panel. Our analysis indicated that these can lead to misidentifications or overrepresentation of ancestry components and selective traits in imputed genotypes. This is especially significant considering the weight archaeological debates place on ancestral relationships and admixture patterns of domesticated animals to understand the mechanisms of emergence and dispersal of early animal husbandry throughout the Neolithic across Europe and the Near East. This further highlights the measures needed to interpret the results and biases introduced by imputation and difficulty of imputation of admixed individuals. A more diverse reference panel is one of the most important priorities in ancient *Sus* imputation and particularly, introducing diversity present in ancient *Sus* could elevate accuracy and limit bias.

## Data Availability

Publicly available datasets were analyzed in this study. These data can be found here: https://www.ebi.ac.uk/ena/browser/view/PRJEB30282?show=reads Accession number PRJEB30282.
