# Supplementary material for: Imputation of Ancient Whole Genome Sus scrofa DNA Introduces Biases Toward Main Population Components in the Reference Panel
Source: Front Genet. 2022 Jul 12;13:872486. doi: 10.3389/fgene.2022.872486 (PMC9315352; doi:10.3389/fgene.2022.872486)
Supplement: Supplementary file 3 [file Table1.docx]

# Supplementary Methods

## Subsets of reference panel

Imputation was performed on subsets of the original reference panel to assess the influence of geographical and/or status (wild or domestic) on imputation performance. These subsets consisted of Dutch + French wild boar, Italian wild boar, European domestic pigs, Greece wild boar and Near Eastern + Turkish wild boar. They did decreased genotype concordance between imputed and High-Quality (HQ) genotypes. However, this decrease was most noticeable between distinct geographical origins, e.g. Near Eastern + Turkish wild boar as reference panel and ancient European *Sus* as downsampled target sample. The subsets did not have enough power for accurate imputation, because the reference panel is too limited to allow for subdivision. The original reference panel had the highest genotype concordance and was used as the main reference panel.

## Joined imputation

Imputation was performed on joined VCF files and individual VCF files to assess imputation performance between the two approaches. Two joined VCF files were tested: 1) with only the target samples for imputation (KD033, KD037, VEM185); and 2) all ancient samples (KD033, KD037, VEM185, KD025, AL718, AA119. AA288, AA451, AA363). The joined VCF files were phased prior to imputation with Beagle5, with default parameters apart from changing the effective population size (Ne) to 20,000. Imputation was performed in the same way as the individual imputation. Genotype concordance of the joined imputation was calculated in two different ways; 1) calculating the fraction of genotypes that were imputed correctly/incorrectly between the joined imputed genotypes and the joined HQ genotypes and, 2) retrieving the genotypes from the target samples (KD033, KD037, VEM185) and comparing them to their respective HQ genotypes. Both ways resulted in lower genotype concordance than individual imputation, which might be attested to the diversity of the ancient samples.

## Chromosomal imputation

Imputation was performed for chromosome 1-18 individually, within the chromosomal imputation a variety of sliding windows were tested. The sliding windows consisted of segments of 50,000, 40,000, 30,000 and 20,000 markers with an overlap of 25,000, 20,000, 15,000 and 10,000 markers, respectively. The target VCF files were split into subsets on the amount of respective markers and overlapped with splitvcf.jar from Beagle5. For each individual segment genotypes were imputed, combined and overlapping markers were removed. This resulted in autosome wide genotype concordance for each respective sliding window subset. The sliding window approach resulted in lower genotype concordance compared to whole chromosome imputation. Imputation on whole chromosomes was used to test imputation accuracy

## Known genomic positions

We used known genomic positions as a filter for imputed genotypes to assess their contribution to genotype concordance. Three different datasets with known genomic positions were used: 1) 50k porcine SNP-Chip (BRON); 2) the transversions only; and 3) main SNP-sets from the study of Frantz *et al*. 2019. These datasets were mapped onto the *Sus scrofa* 10.2 reference genome and were lifted over to the *Sus scrofa* 11.1 reference (used in this study) with CrossMap v0.5.2 using the susScr3ToSusScr11.over.chain.gz (https://hgdownload.soe.ucsc.edu/goldenPath/susScr3/liftOver/). The confident positions were used as known genomic positions, whereas all imputed genotypes falling out of these sites were filtered.

## Downsampling KD037 and VEM185

KD037 and VEM185 were downsampled to match the coverage of KD033, to test if the difference in coverage between the samples leads to differences in genotype concordance. KD037 and VEM185 were down sampled to 6.9x, and further downsampled to 1x for imputation, both with Picard v2.18.17. The same imputation pipeline was used for these two times downsampled samples. There was no significant difference in genotype concordance between the original and downsampled (6.7x) samples.

## Including Asian samples

To test the effect of potential bias towards Asian haplotypes sixteen Asian domestic pigs, 6 Asian wild boar and 3 South-East Asian outgroups were added to the reference panel (Supplementary Table 1). Target individuals were newly downsampled, resulting in a deviation between original genotype concordances. Including Asian samples had little to no effect on genotype concordance for Beagle5 (Including Asian samples-Figure 1). Genotype concordance for Impute5 was only calculated for a couple of test chromosomes due to the increased memory usage of the added samples. This resulted in a similar trend as in Beagle5, where none to little difference between genotype concordance was observed. However, the addition of Asian samples introduced a bias in downstream analyses (Including Asian samples-Figure 2). Therefore, it was decided to remove Asian samples from the reference panel to mitigate any potential bias.


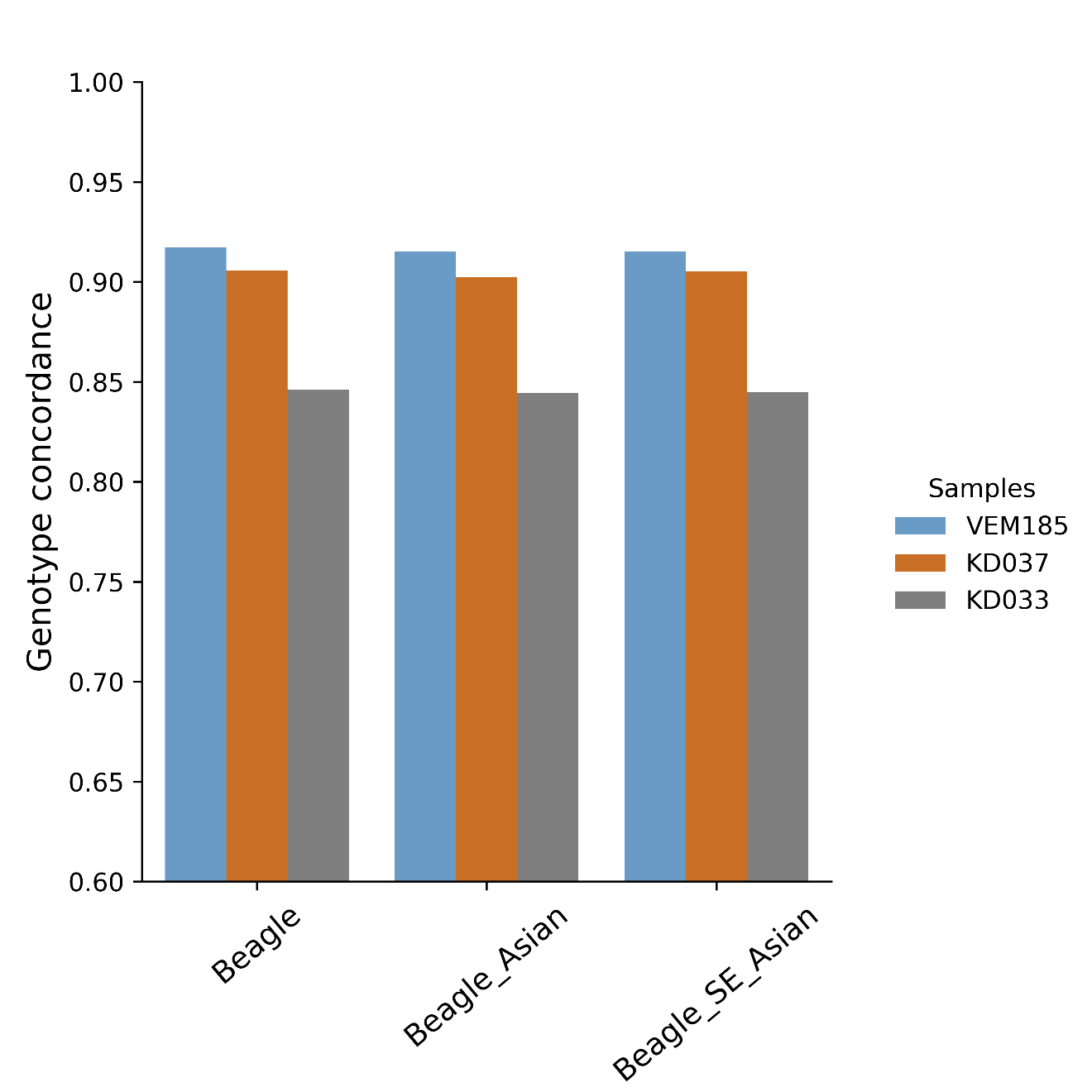


Figure 1-Including Asian samples. Genotype concordance (portrayed in decimals using Beagle with additions of Asian samples. Imputed from 1x downsampled coverage genomes of VEM185, KD037 and KD033.


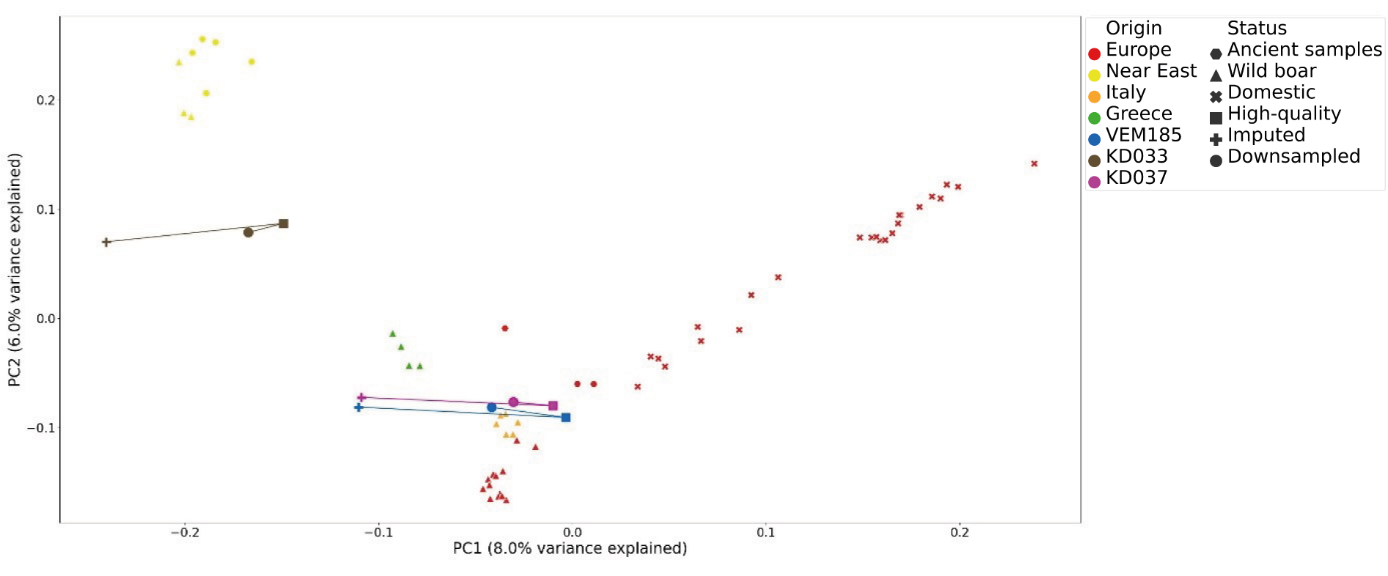


Figure 2-Including Asian Samples. PCA comparing HQ genotyped with Imputed genotypes. Imputed samples were imputed with Asian samples in the reference panel.

# Supplementary Tables & Figures

Table 1. Sample specifics

| ID | Population | Breed/Country | Coverage | Accession | doi | Source |
| --- | --- | --- | --- | --- | --- | --- |
| AS01F01 | EUD | Angler_Sattleschwein | 11.9 | SAMEA3497824 | 10.1038/ng.3394 | Wageningen |
| AS01F09 | EUD | Angler_Sattleschwein | 10.01 | SAMEA3497825 | 10.1038/ng.3394 | Wageningen |
| BK01F10 | EUD | Berkshire | 12.01 | SAMEA3497827 | 10.1038/ng.3394 | Wageningen |
| BK01M20 | EUD | Berkshire | 10.18 | SAMEA3497828 | 10.1038/ng.3394 | Wageningen |
| BB01M47 | EUD | Bunte_Bentheimer | 14.4 | SAMEA3497826 | 10.1038/ng.3394 | Wageningen |
| CA01F14 | EUD | Calabrese | 11.74 | SAMEA3497831 | 10.1038/ng.3394 | Wageningen |
| CT01F13 | EUD | Casertana | 11.15 | SAMEA3497835 | 10.1038/ng.3394 | Wageningen |
| CT01M12 | EUD | Casertana | 8.71 | SAMEA3497836 | 10.1038/ng.3394 | Wageningen |
| CM01F17 | EUD | Chato_Murciano | 10.86 | SAMEA3497832 | 10.1038/ng.3394 | Wageningen |
| CM01F18 | EUD | Chato_Murciano | 9.39 | SAMEA3497833 | 10.1038/ng.3394 | Wageningen |
| CS01F02 | EUD | Cinta_Senese | 10.79 | SAMEA3497834 | 10.1038/ng.3394 | Wageningen |
| WB21F03 | EUW | Dutch_wild1 | 10.75 | SAMEA3497864 | 10.1038/ng.3394 | Wageningen |
| WB21F04 | EUW | Dutch_wild1 | 12.8 | SAMEA3497865 | 10.1038/ng.3394 | Wageningen |
| WB21F05 | EUW | Dutch_wild1 | 9.37 | SAMEA1557424 | 10.1371/journal.pgen.1003100 | Wageningen |
| WB21M03 | EUW | Dutch_wild1 | 10.17 | SAMEA1557433 | 10.1371/journal.pgen.1003100 | Wageningen |
| WB21M05 | EUW | Dutch_wild1 | 18.73 | SAMEA3497869 | 10.1038/ng.3394 | Wageningen |
| WB22F01 | EUW | Dutch_wild1 | 6.42 | [SAMEA1557394](https://www.ebi.ac.uk/ena/data/view/SAMEA1557394) | [SAMEA3497870](https://www.ebi.ac.uk/ena/data/view/SAMEA3497870) | Wageningen |
| WB22F02 | EUW | Dutch_wild2 | 7.9 | SAMEA1557387 | 10.1371/journal.pgen.1003100 | Wageningen |
| WB22F03 | EUW | Dutch_wild2 | 9.46 | SAMEA3497872 | 10.1038/ng.3394 | Wageningen |
| WB22F04 | EUW | Dutch_wild2 | 9 | SAMEA3497873 | 10.1038/ng.3394 | Wageningen |
| WB22M03 | EUW | Dutch_wild2 | 12.07 | SAMEA3497875 | 10.1038/ng.3394 | Wageningen |
| WB25U11 | EUW | French_wild | 9.67 | SAMEA1557401 | 10.1371/journal.pgen.1003100 | Wageningen |
| GO01F04 | EUD | Gloucester_Old_Spot | 16.37 | SAMEA3497841 | 10.1038/ng.3394 | Wageningen |
| GO01F23 | EUD | Gloucester_Old_Spot | 9.09 | SAMEA3497842 | 10.1038/ng.3394 | Wageningen |
| WB31F05 | BLW | Greece1 | 10.62 | SAMEA3497880 | 10.1038/ng.3394 | Wageningen |
| WB31M09 | BLW | Greece1 | 9.35 | SAMEA3497881 | 10.1038/ng.3394 | Wageningen |
| WB32F07 | BLW | Greece2 | 10.1 | SAMEA3497882 | 10.1038/ng.3394 | Wageningen |
| WB32U05 | BLW | Greece2 | 9.58 | SAMEA3497883 | 10.1038/ng.3394 | Wageningen |
| WB44U06 | EUW | Italian_wild | 12.68 | SAMEA3497887 | 10.1038/ng.3394 | Wageningen |
| WB44U07 | EUW | Italian_wild | 11.53 | SAMEA3497888 | 10.1038/ng.3394 | Wageningen |
| WB28F31 | EUW | Italy1 | 15.85 | SAMEA3497878 | 10.1038/ng.3394 | Wageningen |
| WB28M39 | EUW | Italy1 | 11.6 | SAMEA3497879 | 10.1038/ng.3394 | Wageningen |
| WB42M09 | EUW | Italy2 | 13.12 | SAMEA3497886 | 10.1038/ng.3394 | Wageningen |
| LB01F49 | EUD | Large_Black | 18.6 | SAMEA3497845 | 10.1038/ng.3394 | Wageningen |
| LE01F25 | EUD | Leicoma | 12.04 | SAMEA3497846 | 10.1038/ng.3394 | Wageningen |
| LS01F04 | EUD | Linderodsvin | 11.83 | SAMEA3497852 | 10.1038/ng.3394 | Wageningen |
| MA01F18 | EUD | Mangalica | 9.33 | SAMEA3497854 | 10.1038/ng.3394 | Wageningen |
| MA01F20 | EUD | Mangalica | 10.57 | SAMEA3497855 | 10.1038/ng.3394 | Wageningen |
| WB72U01 | NEW | Near_East | 4.4 | SAMEA3497889 | 10.1038/ng.3394 | Wageningen |
| NI01U07 | EUD | Negro_Iberico | 11.84 | SAMEA3497858 | 10.1038/ng.3394 | Wageningen |
| NS01F05 | EUD | Nera_Siciliana | 6.73 | SAMEA3497859 | 10.1038/ng.3394 | Wageningen |
| WB21F10 | EUW | Netherlands | 12.19 | SAMEA3497867 | 10.1038/ng.3394 | Wageningen |
| WB22M02 | EUW | Netherlands | 13.86 | SAMEA3497874 | 10.1038/ng.3394 | Wageningen |
| RE01F51 | EUD | Retinto | 9.82 | SAMEA3497861 | 10.1038/ng.3394 | Wageningen |
| WB33U04 | NEW | Samos_wild | 11.8 | SAMEA3497884 | 10.1038/ng.3394 | Wageningen |
| WB33U05 | NEW | Samos_wild | 10.72 | SAMEA3497885 | 10.1038/ng.3394 | Wageningen |
| WB26M09 | EUW | Swiss_wild | 8.8 | SAMEA1557403 | 10.1371/journal.pgen.1003100 | Wageningen |
| TA01F19 | EUD | Tamworth | 11.05 | SAMEA3497862 | 10.1038/ng.3394 | Wageningen |
| TA01M06 | EUD | Tamworth | 12.56 | SAMEA3497863 | 10.1038/ng.3394 | Wageningen |
| IBGM0327 | EUD | Iberian | 10.5 | SAMN02904857 | 10.1038/hdy.2014.81 | Ramírez et al |
| WBES0717 | EUW | Spain_wild | 12.5 | SAMN02904855 | 10.1038/hdy.2014.81 | Ramírez et al |
| DU23M01 | ASD | Duroc | 10.65 | ERP001813 | [10.1371/journal.pgen.1003100](https://doi.org/10.1371/journal.pgen.1003100) | Wageningen |
| DU23M04 | ASD | Duroc | 8.3 | ERP001813 | [10.1371/journal.pgen.1003100](https://doi.org/10.1371/journal.pgen.1003100) | Wageningen |
| HA20U01 | ASD | Hampshire | 12.3 | ERP001813 | [10.1371/journal.pgen.1003100](https://doi.org/10.1371/journal.pgen.1003100) | Wageningen |
| HA20U02 | ASD | Hampshire | 9.8 | ERP001813 | [10.1371/journal.pgen.1003100](https://doi.org/10.1371/journal.pgen.1003100) | Wageningen |
| LR21M03 | ASD | Landrace | 10.4 | ERP001813 | [10.1371/journal.pgen.1003100](https://doi.org/10.1371/journal.pgen.1003100) | Wageningen |
| LR30F03 | ASD | Landrace | 7.8 | ERP001813 | [10.1371/journal.pgen.1003100](https://doi.org/10.1371/journal.pgen.1003100) | Wageningen |
| LW22M04 | ASD | Large White | 9.8 | ERP001813 | [10.1371/journal.pgen.1003100](https://doi.org/10.1371/journal.pgen.1003100) | Wageningen |
| LW22M07 | ASD | Large White | 10.8 | ERP001813 | [10.1371/journal.pgen.1003100](https://doi.org/10.1371/journal.pgen.1003100) | Wageningen |
| PI21F06 | ASD | Pietrain | 10.9 | ERP001813 | [10.1371/journal.pgen.1003100](https://doi.org/10.1371/journal.pgen.1003100) | Wageningen |
| PI21M20 | ASD | Pietrain | 6 | ERP001813 | [10.1371/journal.pgen.1003100](https://doi.org/10.1371/journal.pgen.1003100) | Wageningen |
| JQ01U02 | ASD | Jianquhai | 11.2 | ERP001813 | [10.1371/journal.pgen.1003100](https://doi.org/10.1371/journal.pgen.1003100) | Wageningen |
| MS20U10 | ASD | Meishan | 9.3 | ERP001813 | [10.1371/journal.pgen.1003100](https://doi.org/10.1371/journal.pgen.1003100) | Wageningen |
| MS20U11 | ASD | Meishan | 9.2 | ERP001813 | [10.1371/journal.pgen.1003100](https://doi.org/10.1371/journal.pgen.1003100) | Wageningen |
| MS21M14 | ASD | Meishan | 11.2 | ERP001813 | [10.1371/journal.pgen.1003100](https://doi.org/10.1371/journal.pgen.1003100) | Wageningen |
| XI01U03 | ASD | Xiang | 9.4 | ERP001813 | [10.1371/journal.pgen.1003100](https://doi.org/10.1371/journal.pgen.1003100) | Wageningen |
| XI01U04 | ASD | Xiang | 9.2 | ERP001813 | [10.1371/journal.pgen.1003100](https://doi.org/10.1371/journal.pgen.1003100) | Wageningen |
| SSWB29U12 | ASW | South China | 10.5 | ERP001813 | [10.1371/journal.pgen.1003100](https://doi.org/10.1371/journal.pgen.1003100) | Wageningen |
| WB29U16 | ASW | South China | 9.5 | ERP001813 | [10.1371/journal.pgen.1003100](https://doi.org/10.1371/journal.pgen.1003100) | Wageningen |
| WB29U04 | ASW | South China | 5.30 | ERP001813 | [10.1371/journal.pgen.1003100](https://doi.org/10.1371/journal.pgen.1003100) | Wageningen |
| SSWB30U08 | ASW | North China | 10.7 | ERP001813 | [10.1371/journal.pgen.1003100](https://doi.org/10.1371/journal.pgen.1003100) | Wageningen |
| WB30U01 | ASW | North China | 4.9 | ERP001813 | [10.1371/journal.pgen.1003100](https://doi.org/10.1371/journal.pgen.1003100) | Wageningen |
| WB30U08 | ASW | North China | 10.5 | ERP001813 | [10.1371/journal.pgen.1003100](https://doi.org/10.1371/journal.pgen.1003100) | Wageningen |
| Sbarbatus | SEA | South-East Asia | 7.1 | ERP001813 | [10.1371/journal.pgen.1003100](https://doi.org/10.1371/journal.pgen.1003100) | Wageningen |
| Scelebensis | SEA | South-East Asia | 25 | ERP001813 | [10.1371/journal.pgen.1003100](https://doi.org/10.1371/journal.pgen.1003100) | Wageningen |
| Sverrucosus | SEA | South-East Asia | 13.4 | ERP001813 | [10.1371/journal.pgen.1003100](https://doi.org/10.1371/journal.pgen.1003100) | Wageningen |
| AA119 | ANC | Armenia_ Lchashen _3050BP | 5.83 | PRJEB30282 | 10.1073/pnas.1901169116 | Ninna Manaseryan |
| AA133 | ANC | Turkmenistan_ Parkhai Karakala _present | 4.5 | PRJEB30282 | 10.1073/pnas.1901169116 | Keith Dobney |
| AA288 | ANC | France_la Baume d’Oulen_7297BP | 4.16 | PRJEB30282 | 10.1073/pnas.1901169116 | Jean-Denis Vigne / Daniel Helmer |
| AA349 | ANC | Iran_ Miankaleh _present | 14.78 | PRJEB30282 | 10.1073/pnas.1901169116 | Marjan Mashkour |
| AA363 | ANC | Iran_ Kohne Tepesi _4492BP | 4.5 | PRJEB30282 | 10.1073/pnas.1901169116 | Marjan Mashkour |
| AA451 | ANC | Faroes_ Undir Junkariusfløtti_950BP | 3 | PRJEB30282 | 10.1073/pnas.1901169116 | Tom McGovern / Mike Church |
| AL718 | ANC | Turkey_ Aşıklı Höyük_10200BP | 7.6 | PRJEB30282 | 10.1073/pnas.1901169116 | Joris Peters |
| KD025 | ANC | Netherlands_Utrecht_2375BP | 5.76 | PRJEB30282 | 10.1073/pnas.1901169116 | Canan Cakirlar |
| VEM185 | ANC, IMP | England_Durrington Wals_6500BP | 21.76 | PRJEB30282 | 10.1073/pnas.1901169116 | Umberto Albarella |
| KD033 | ANC, IMP | Germany_Herxheim_7120BP | 6.88 | PRJEB30282 | 10.1073/pnas.1901169116 | Andrea Zeeb-Lanz |
| KD037 | ANC, IMP | Germany_Herxheim_7120BP | 21.6 | PRJEB30282 | 10.1073/pnas.1901169116 | Andrea Zeeb-Lanz |

Table 2 Filter used for the reference panel and their respective amount of variants

| **ID** | **Filters** | **# of SNPs** |
| --- | --- | --- |
| Main | Repetitive elements | 12,737,362 |
| Main 2-step | Repetitive elements, filter for GL before imputation | 12,737,362 |
| Main Anc | Repetitive elements, ancient samples included | 10,823,257 |
| Main 2-step Anc | Repetitive elements, filter for GL before imputation, ancient samples included | 10,823,257 |
| Main all | Repetitive elements, all confident sites included | 13,388,283 |
| Main 2-step all | Repetitive elements, filter for GL before imputation, all confident sites included | 13,388,283 |
| Main Anc all | Repetitive elements, ancient samples included, all confident sites included | 11,348,166 |
| Main 2-step Anc all | Repetitive elements, filter for GL before imputation, ancient samples included, all confident sites included | 11,348,166 |
| Main 2-step Anc all TS only | Repetitive elements, filter for GL before imputation, ancient samples included, all confident sites included, Transitions only | 7,855,234 |
| Main 2-step Anc all TV only | Repetitive elements, filter for GL before imputation, ancient samples included, all confident sites included, Transversion only | 6,671,529 |
| Main 2-step Anc all MAF <0.05 | Repetitive elements, filter for GL before imputation, ancient samples included, all confident sites included, MAF <0.05 | 4,571,658 |
| Main 2-step Anc all MAF 0.05-0.1 | Repetitive elements, filter for GL before imputation, ancient samples included, all confident sites included, MAF 0.05-0.1 | 1,713,207 |
| Main 2-step Anc all MAF 0.1-0.3 | Repetitive elements, filter for GL before imputation, ancient samples included, all confident sites included, MAF 0.1-0.3 | 2,776,791 |
| Main 2-step Anc all MAF >0.05 | Repetitive elements, filter for GL before imputation, ancient samples included, all confident sites included, MAF >0.05 | 6,638,522 |
| Main 2-step Anc all >0.3 | Repetitive elements, filter for GL before imputation, ancient samples included, all confident sites included, MAF >0.3 | 2,072,318 |

Supplementary Table 3- Genetic map is added as an excel file

Supplementary Table 4- Genotype concordance for all tests is added as an excel file


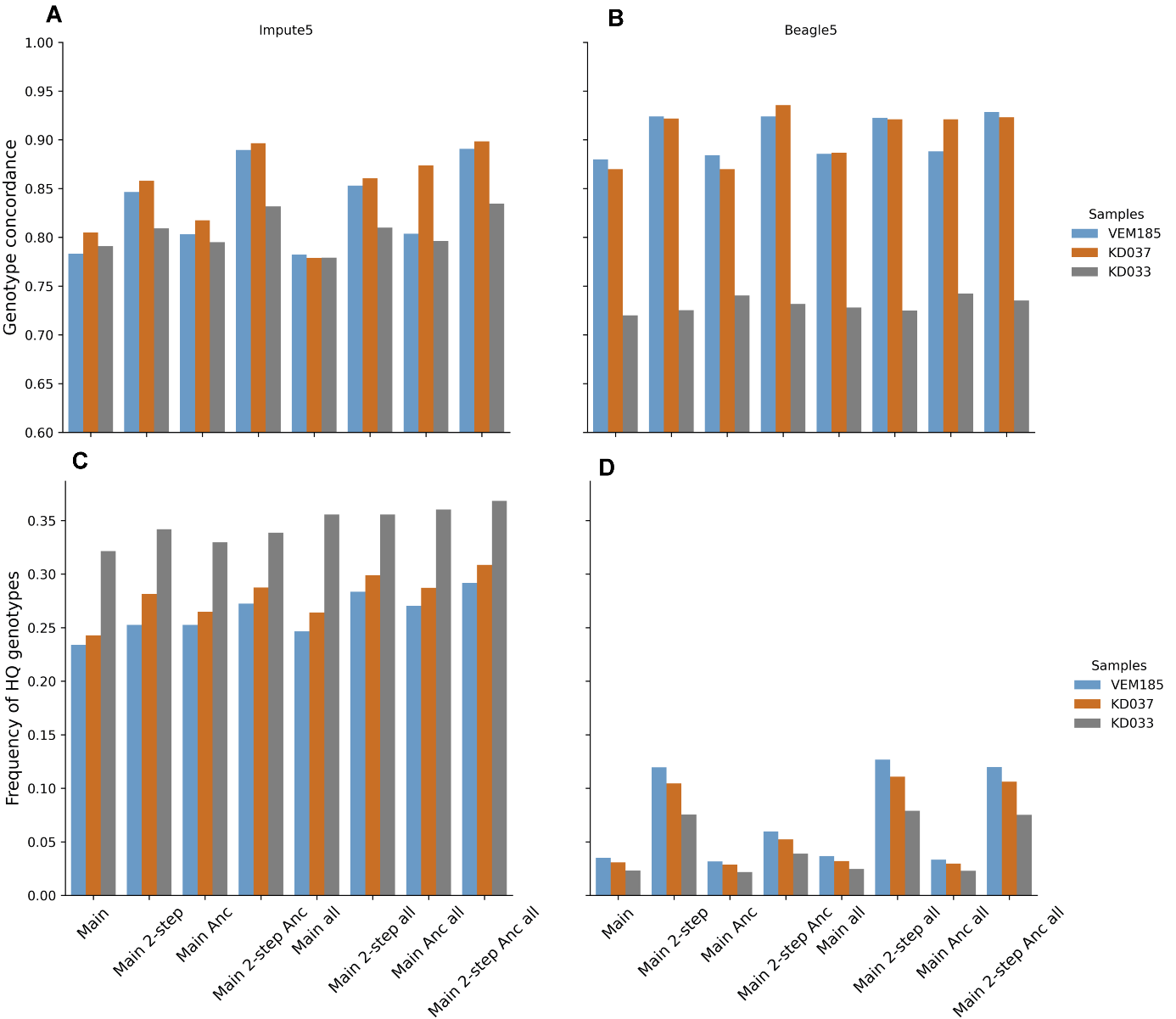


Figure 3. Genotype concordance and Fraction of HQ genotypes (portrayed in decimals, total HQ genotypes: VEM185:4,701,683, KD037: 4,531,126, KD033, 3,887,848) using different imputation tools and filters for the reference panel. Imputed from 1x downsampled coverage genomes of VEM185, KD037 and KD033. Genotype concordance for Impute 5 (A), Beagle5 (B), Fractions of HQ genotypes covered by imputation in Impute5 (C), Beagle5 (D). Main = original one step pipeline, Anc = Ancient samples included, 2-step = new two step pipeline with an extra filtering step, all = all confident sites


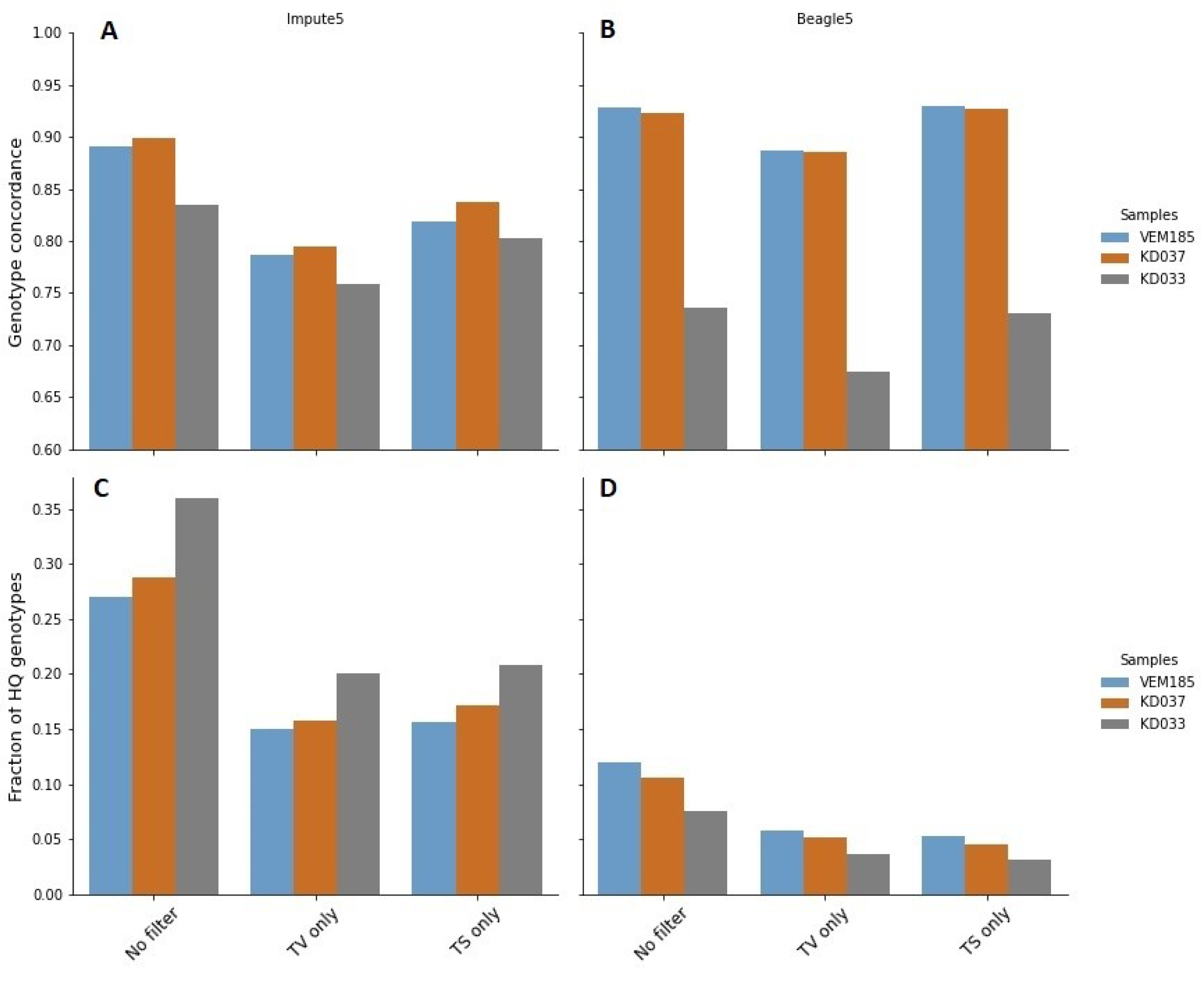


Figure 4. Genotype concordance and Fraction of HQ genotypes (portrayed in decimals, total HQ genotypes: VEM185:4,701,683, KD037: 4,531,126, KD033, 3,887,848) using different imputation tools and filters for the reference panel. Imputed from 1x downsampled coverage genomes of VEM185, KD037 and KD033. Genotype concordance for Impute 5 (A), Beagle5 (B), Fractions of HQ genotypes covered by imputation in Impute5 (C), Beagle5 (D). TV = Transversions, TS = transitions


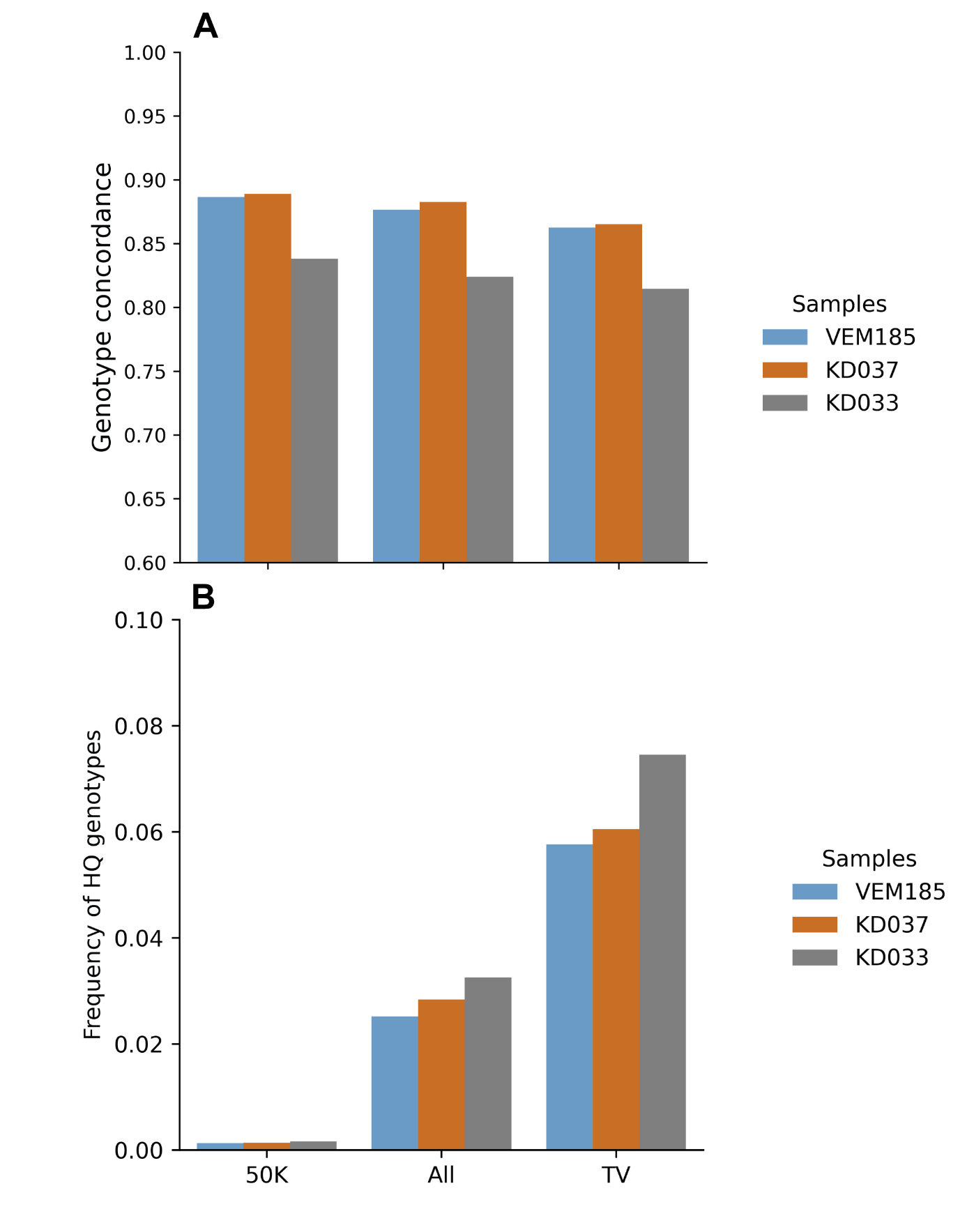


Figure 5. Genotype concordance and Fraction of HQ genotypes (portrayed in decimals, total HQ genotypes: VEM185:4,701,683, KD037: 4,531,126, KD033, 3,887,848) for different known genomic positions. Imputed from a downsampled 1x coverage genomes of VEM185, KD037 and KD033. Genotype concordance for Impute 5 (A), Fractions of HQ genotypes covered by imputation in Impute5 (B). 50K = Porcine SNP-chip, All = Frantz, et al 2019 snpset from high coverage individuals, TV = Frantz et al 2019 Transversions only snpset


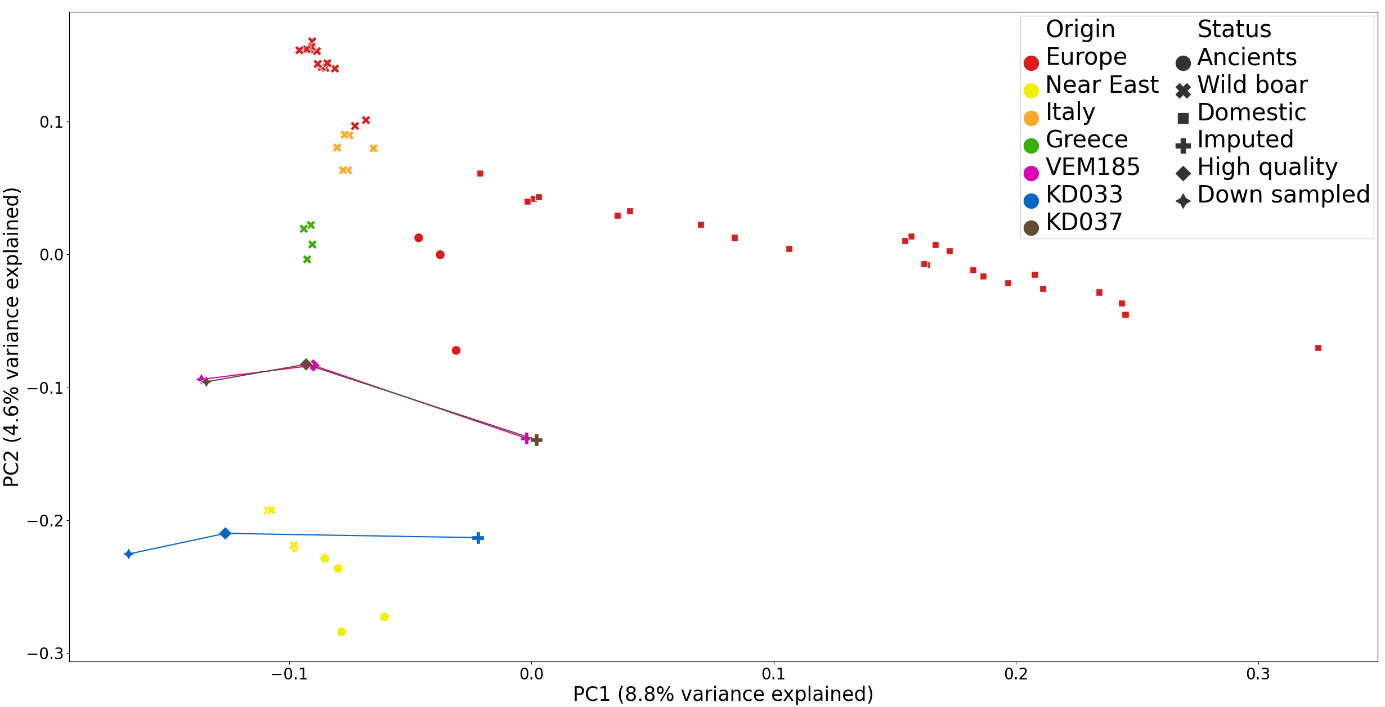


Figure 6. PCA comparing HQ, imputed genotypes and downsampled data together with samples from the reference panel. The imputed samples are from IMP2


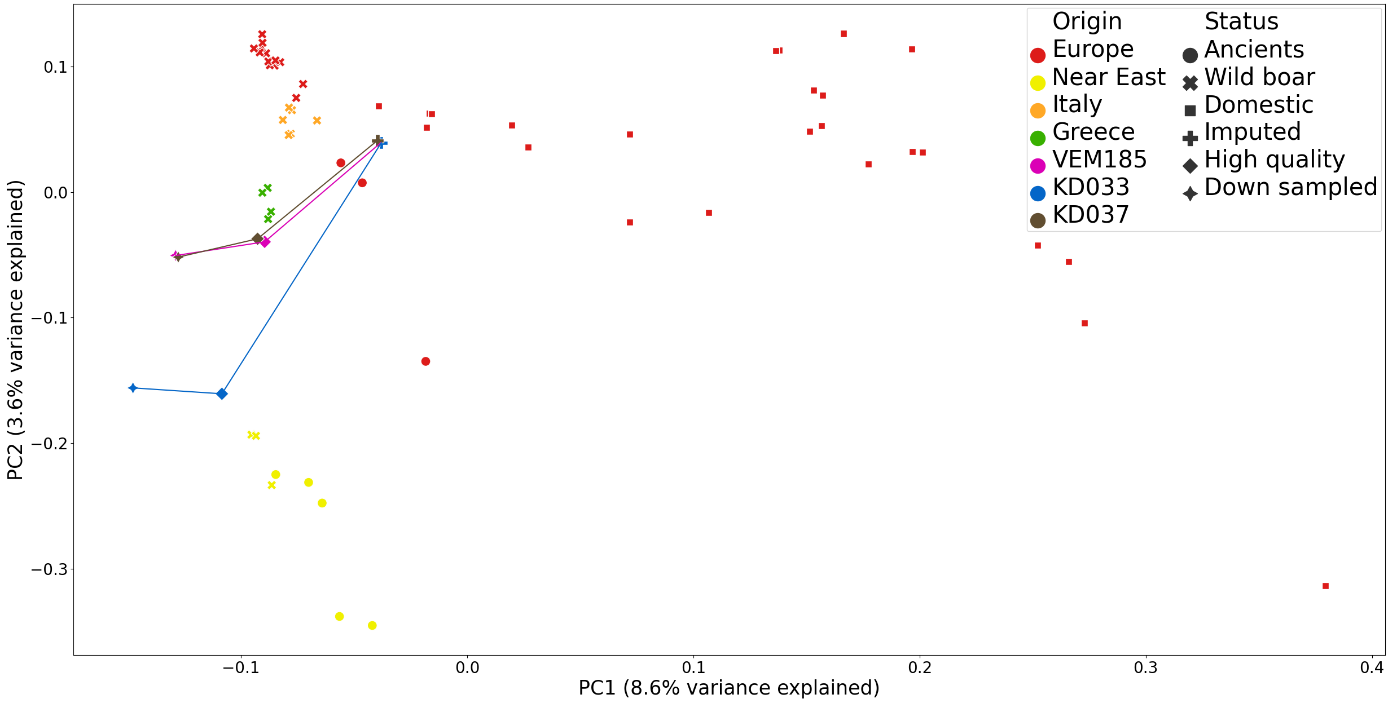


Figure 7. PCA comparing HQ, imputed genotypes and downsampled data together with samples from the reference panel. The imputed samples are from IMP3


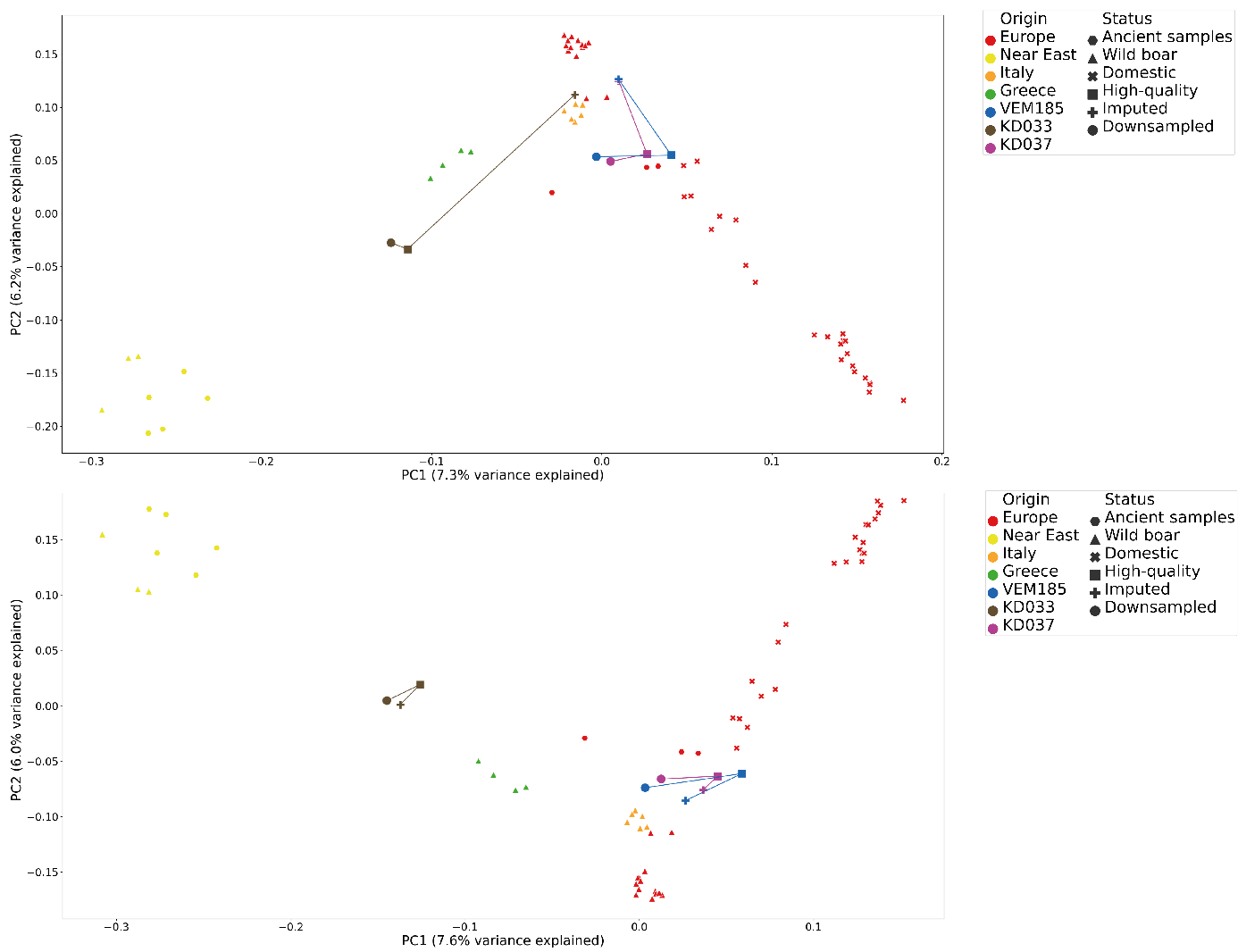


Figure 6. PCA comparing HQ, imputed genotypes and downsampled data together with samples from the reference panel. The imputed samples are from Method 3 using Impute5 (A), and Beagle5 (B).


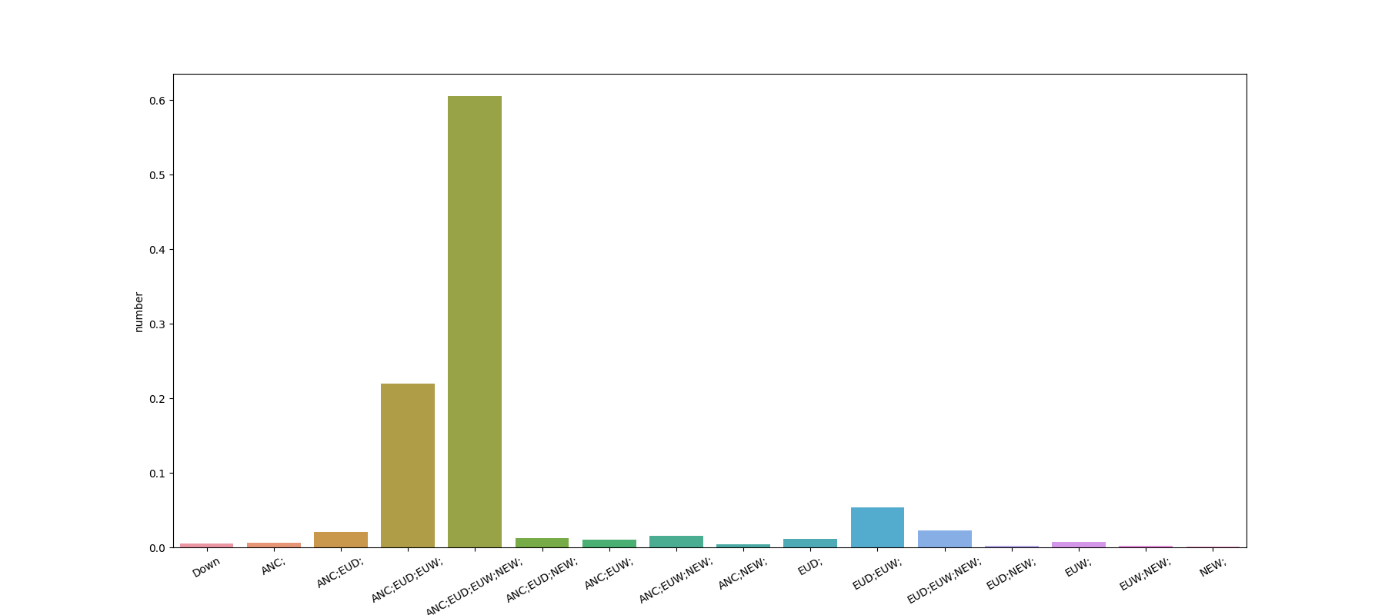

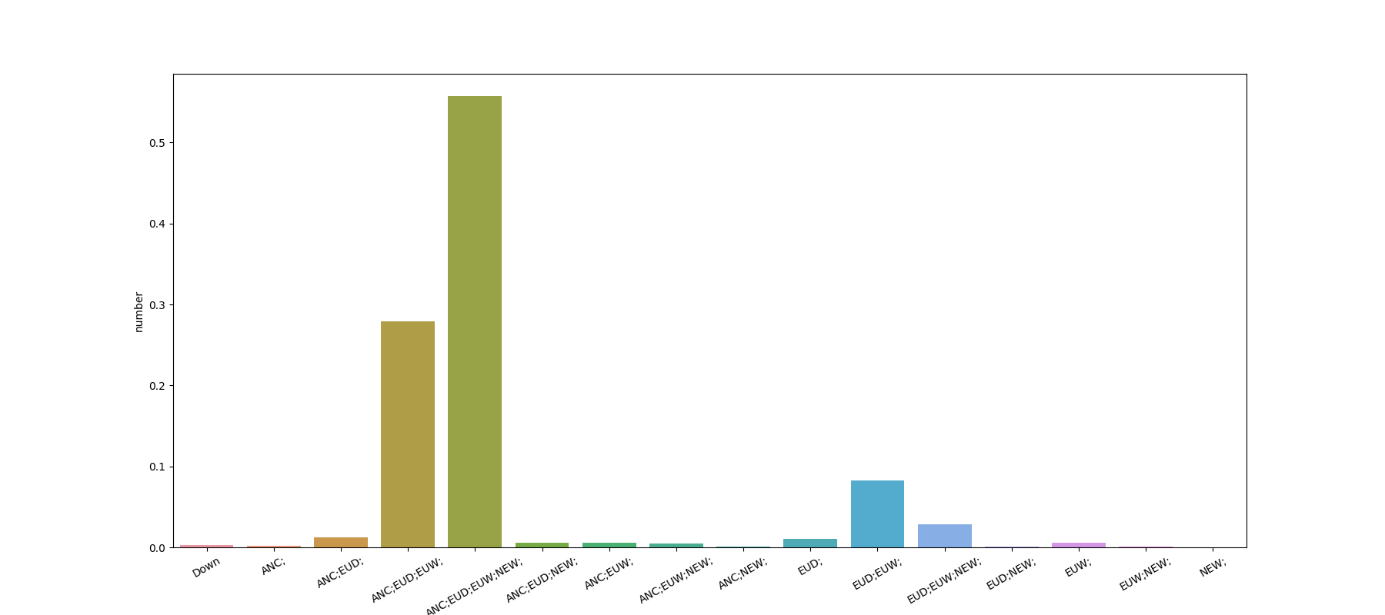

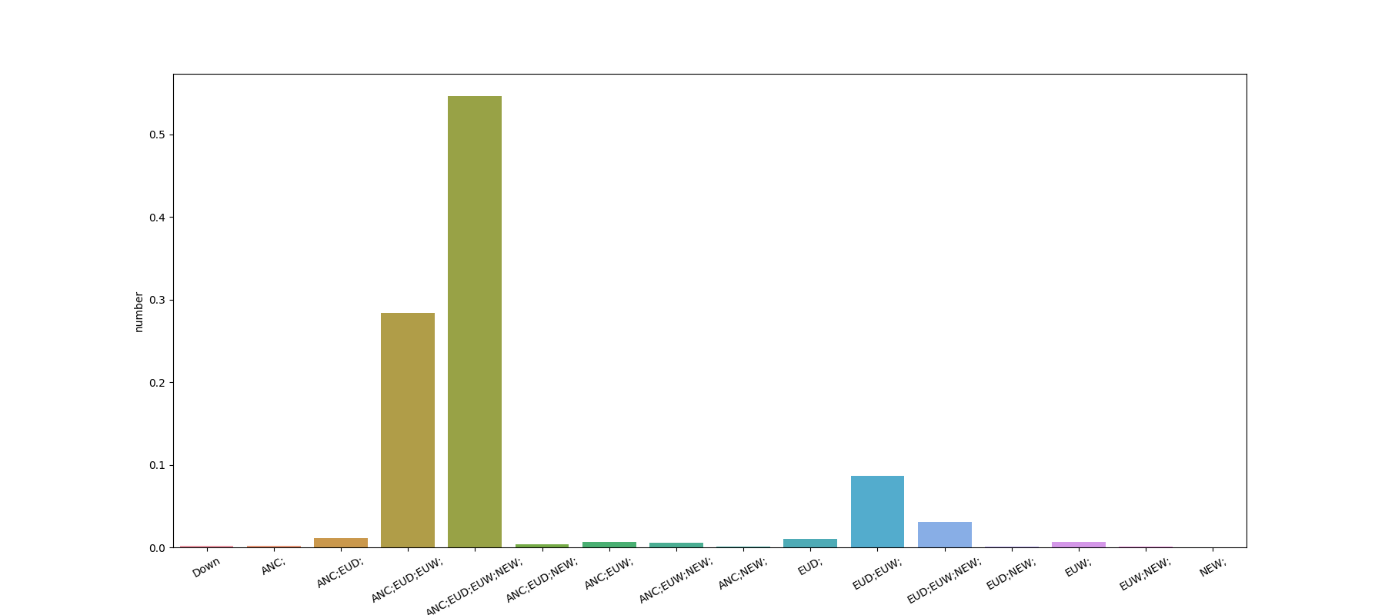


C

B

A

Figure7. Reference bias towards geographical/status groups in the reference panel for the HQ genotypes. (A) KD033, (B) KD037, (C) VEM185.


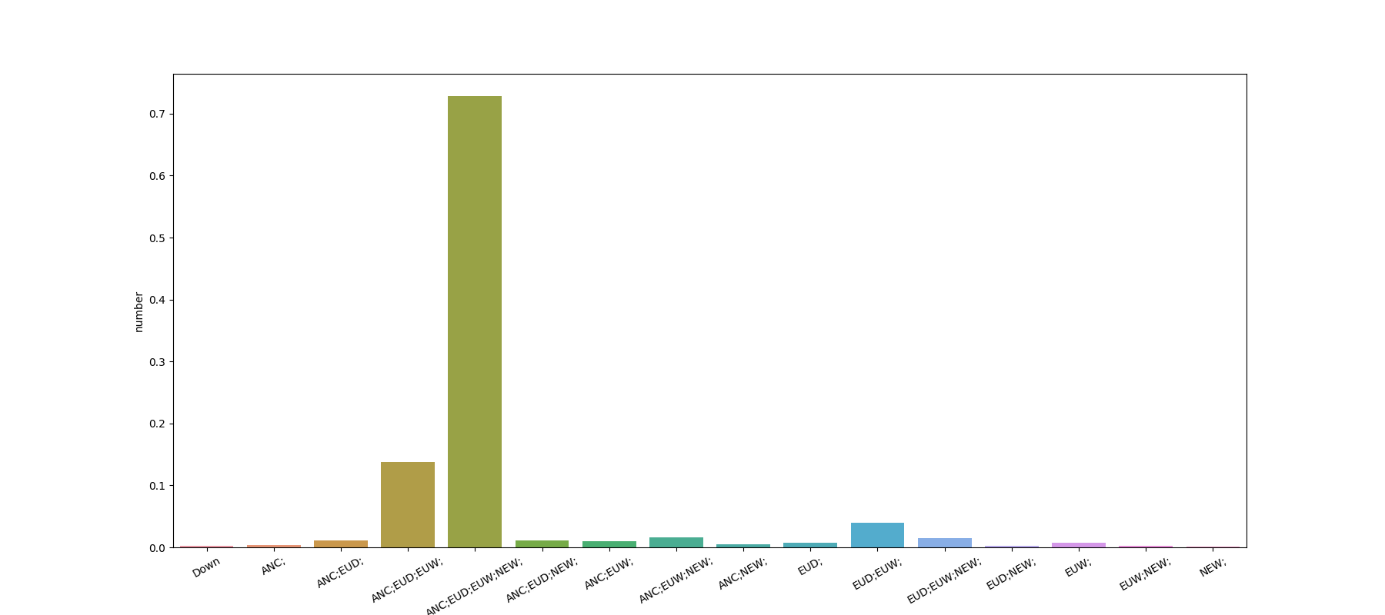

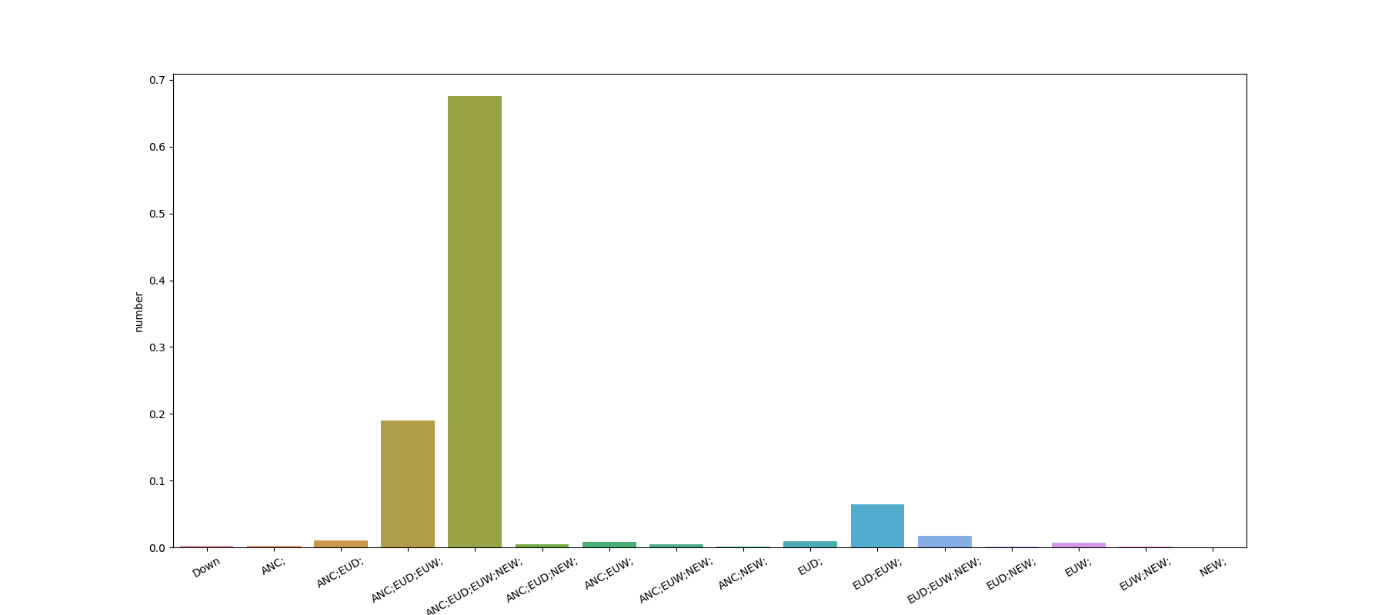

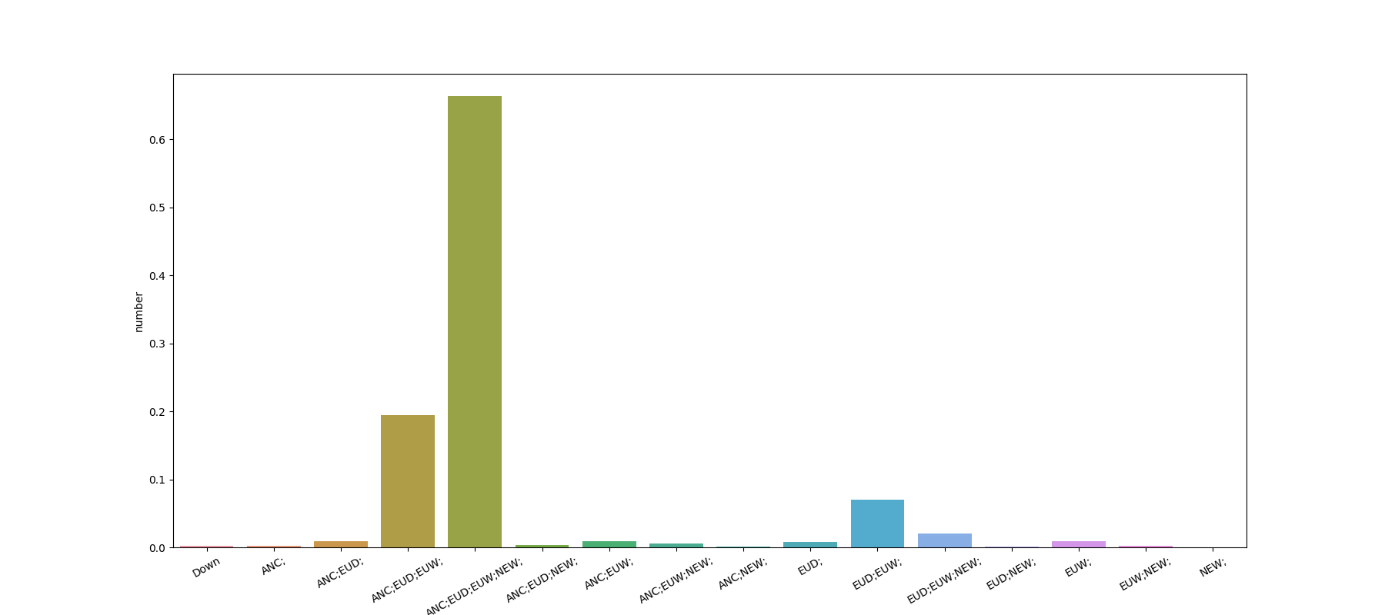


A

B

C

Figure 8. Reference bias towards geographical/status groups in the reference panel for the genotypes imputed by Impute5 that were present in the HQ genotypes (IMP1). (A) KD033, (B) KD037, (C) VEM185.


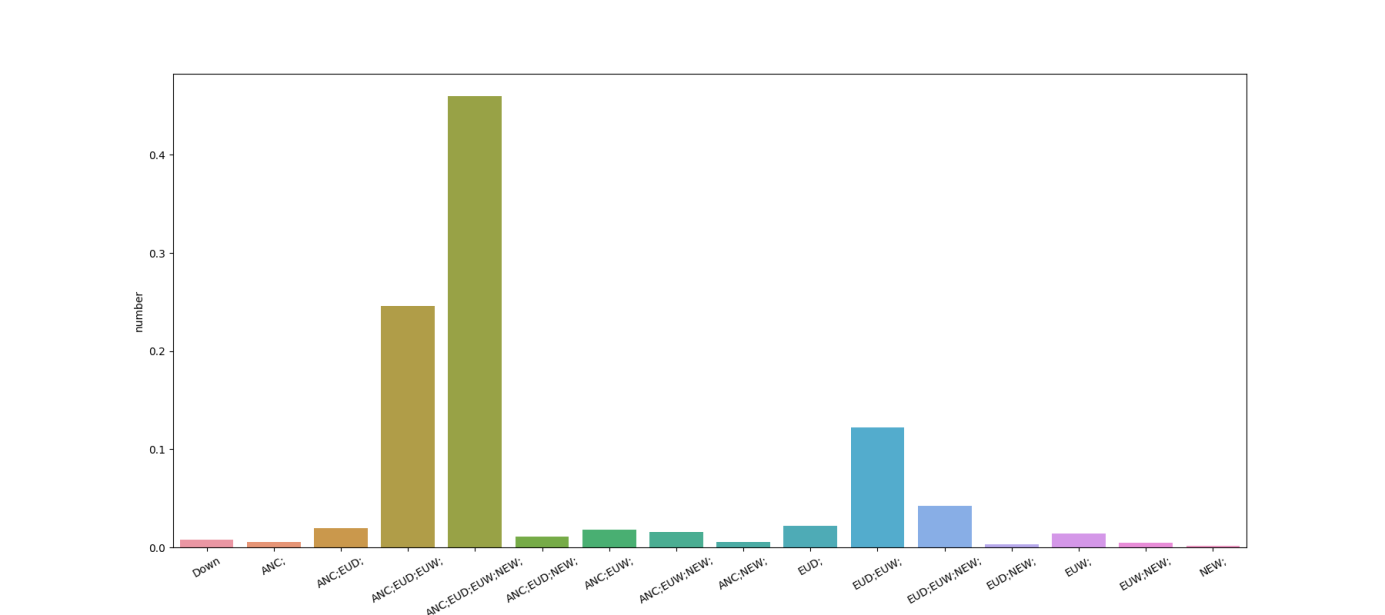

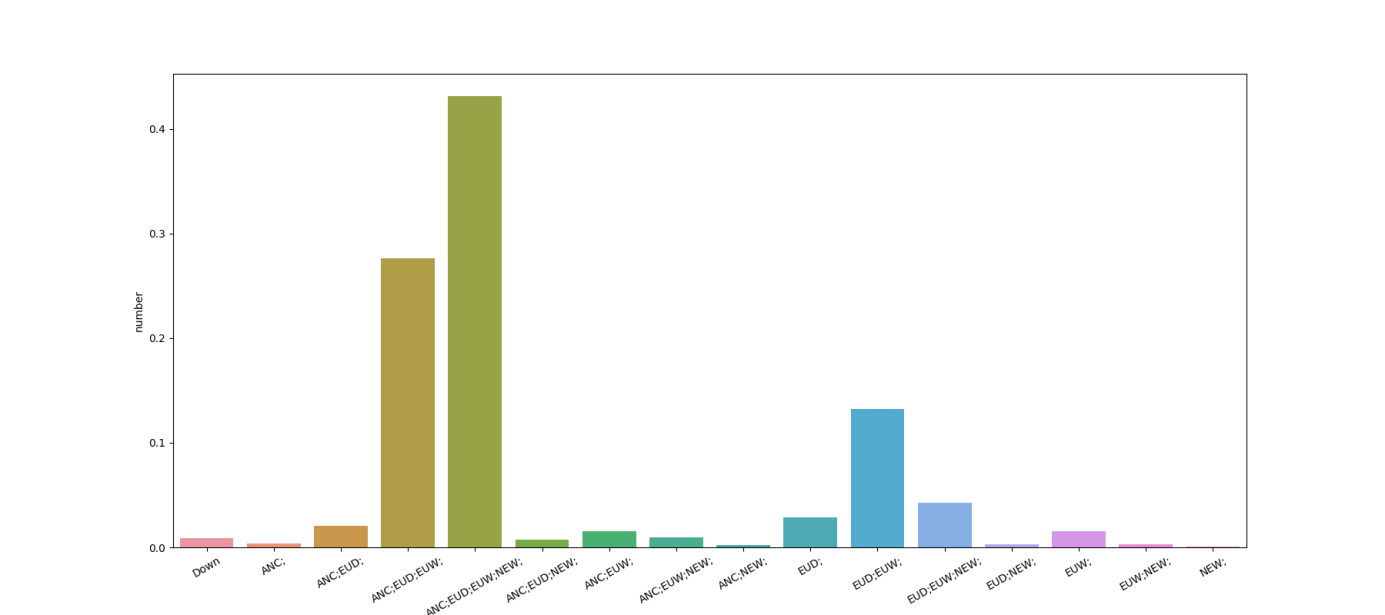

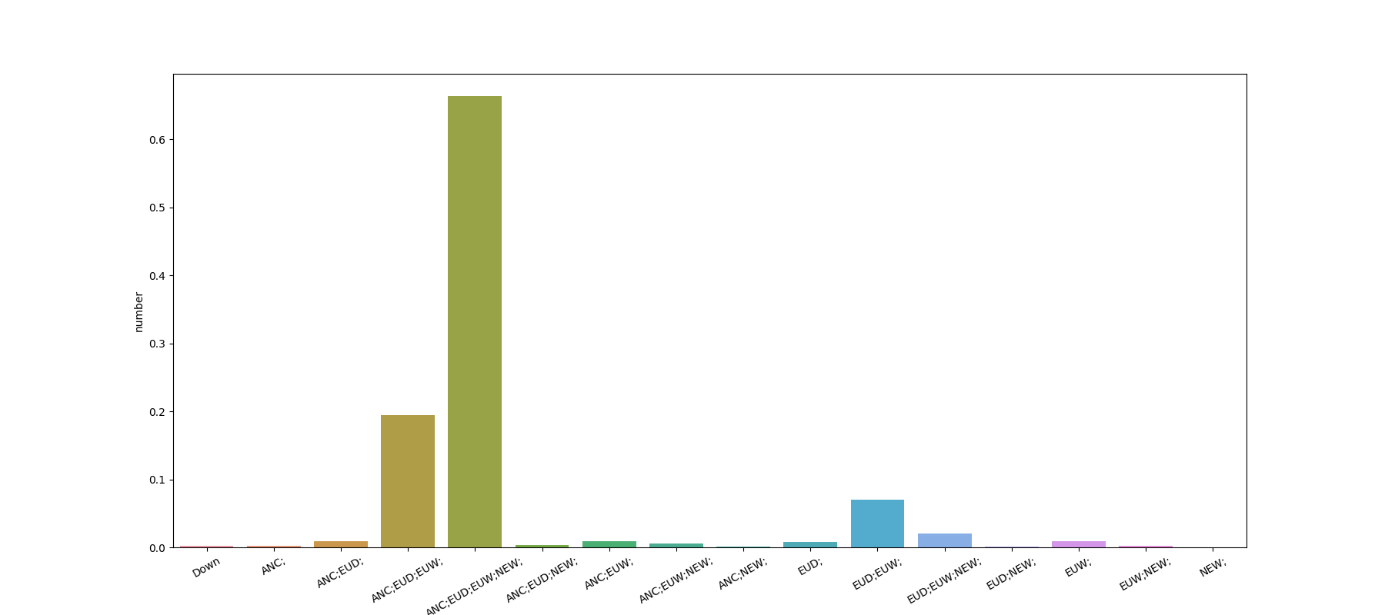


A

B

C

Figure 9. Reference bias towards geographical/status groups in the reference panel for the genotypes imputed by Impute5 that were not present in the HQ genotypes (IMP1). (A) KD033, (B) KD037, (C) VEM185.


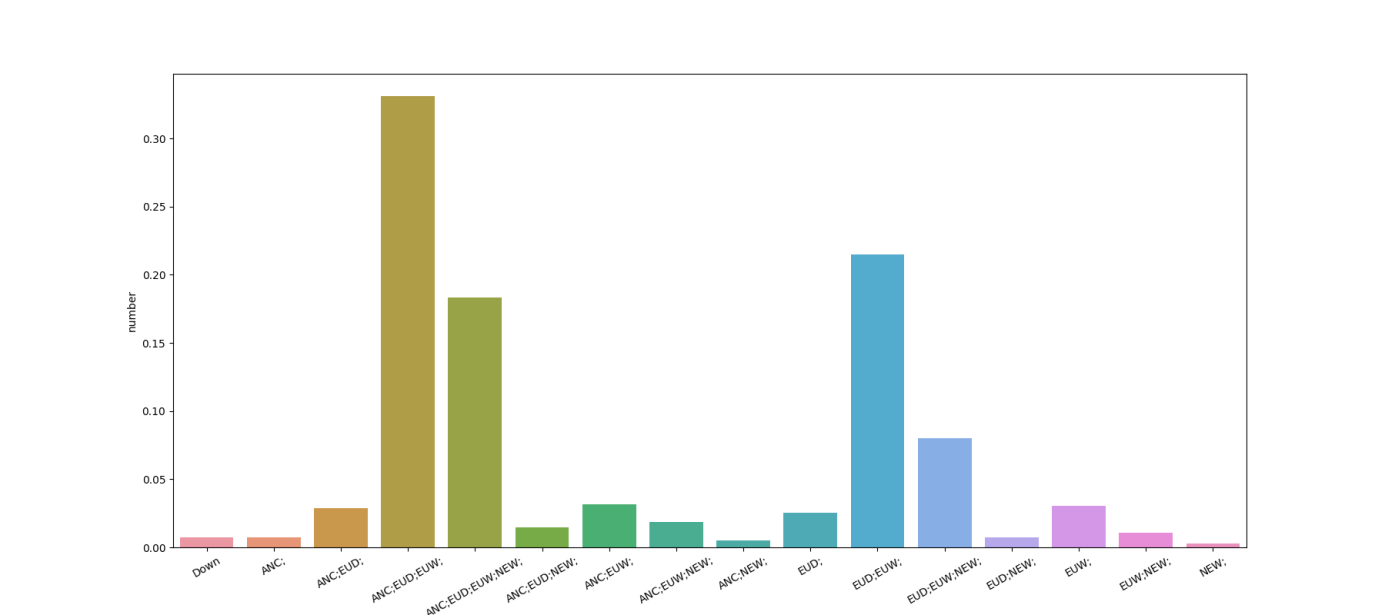

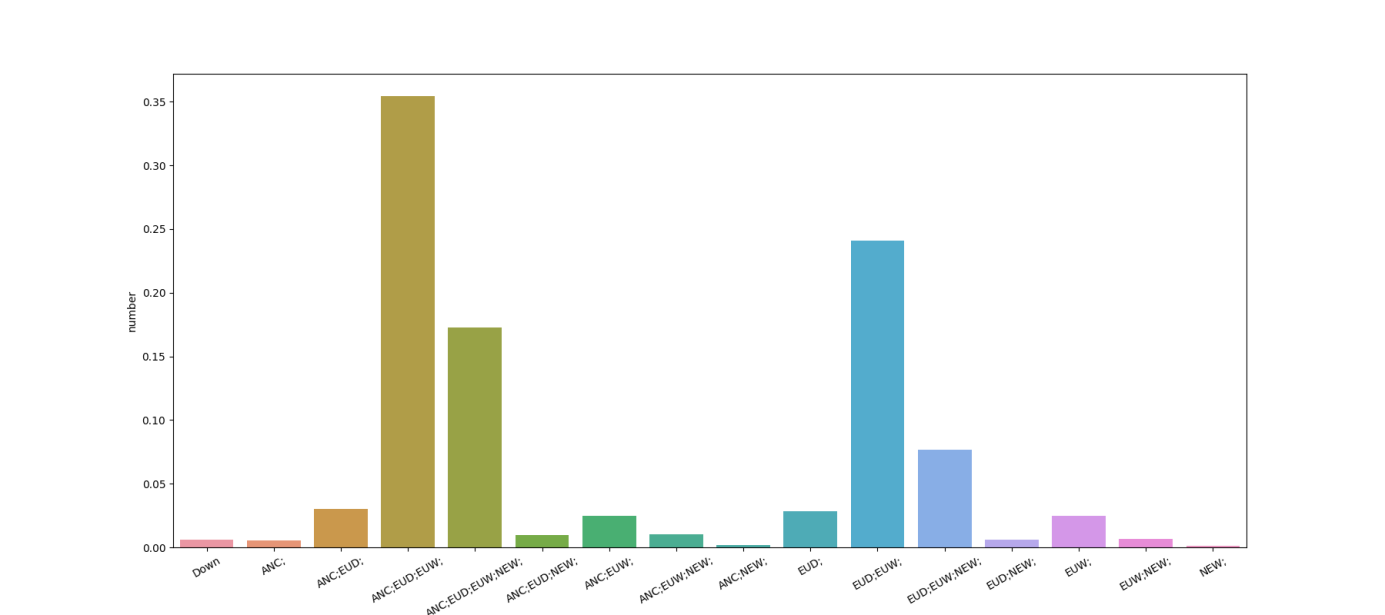

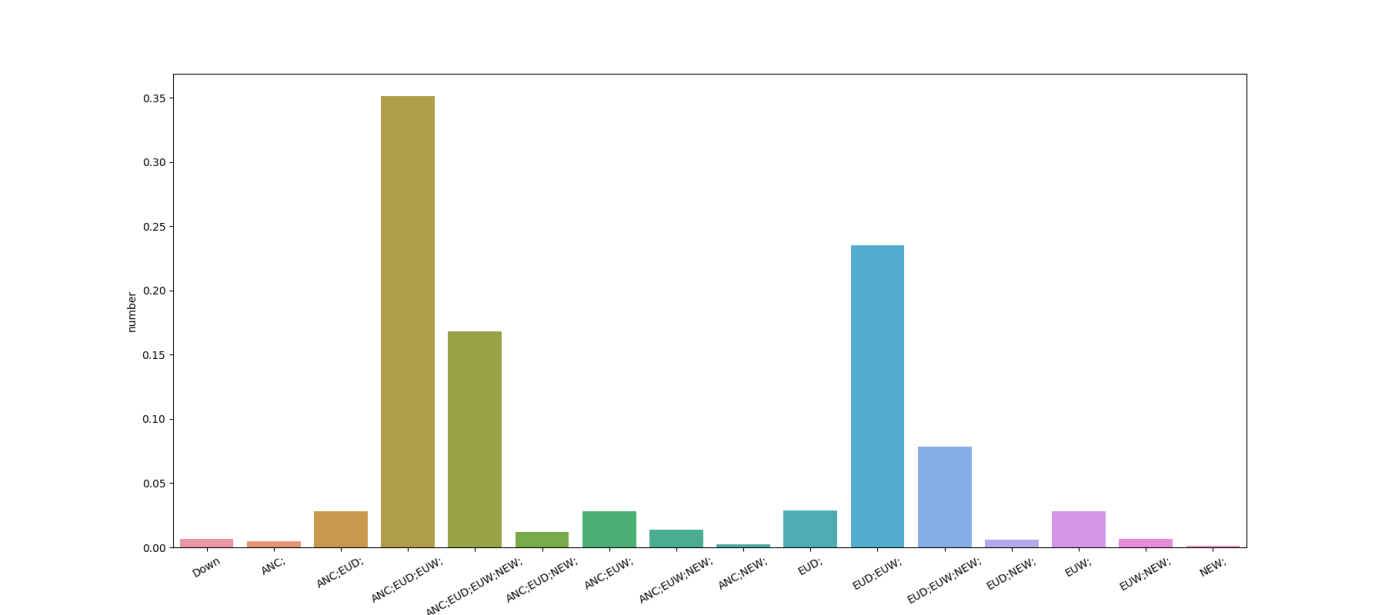


A

B

C

Figure 8. Reference bias towards geographical/status groups in the reference panel for the genotypes imputed by Beagle5 that were present in the HQ genotypes (IMP1). (A) KD033, (B) KD037, (C) VEM185.


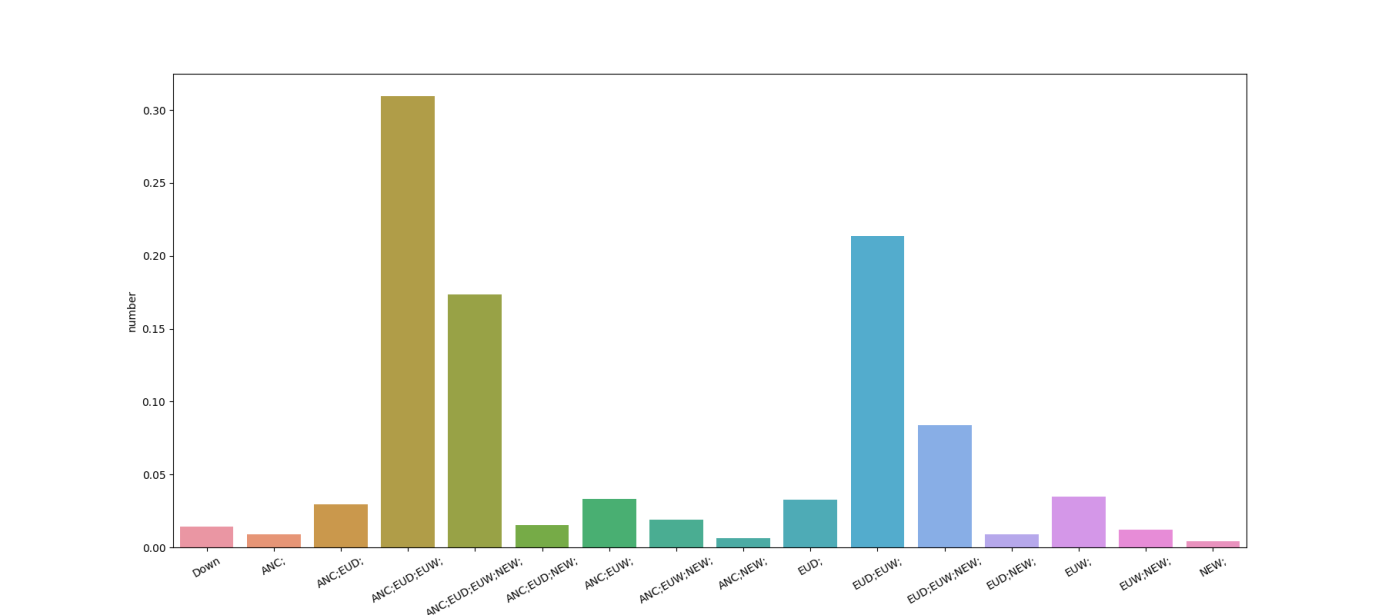

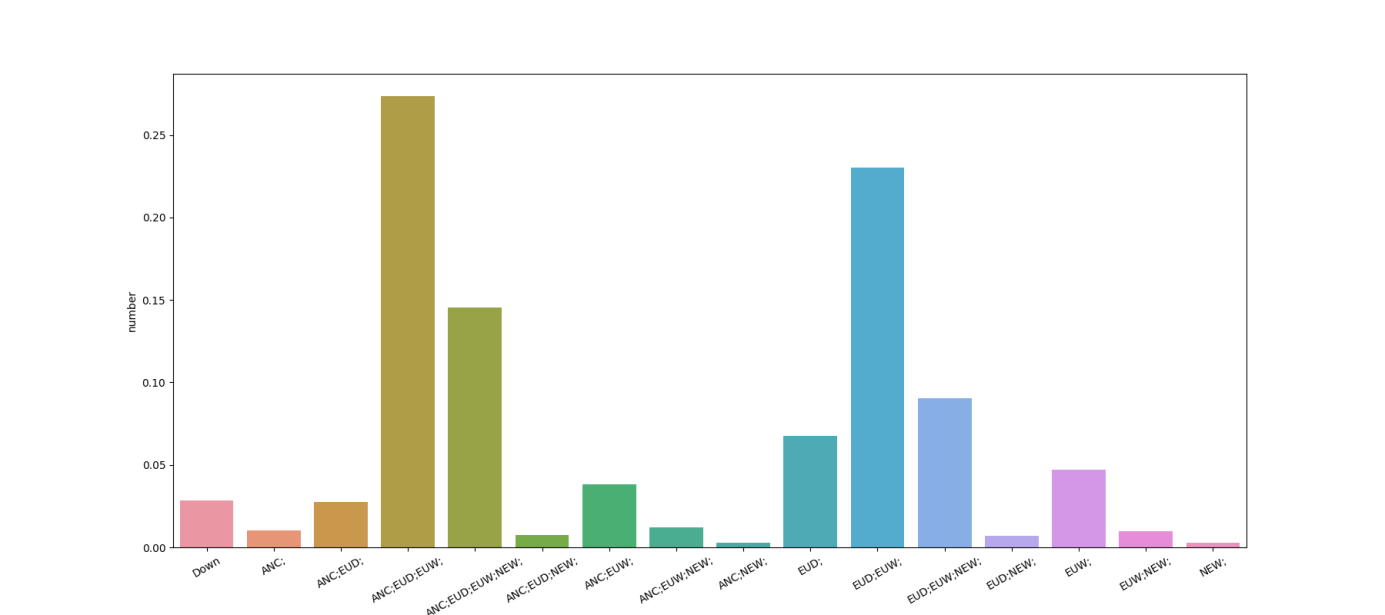

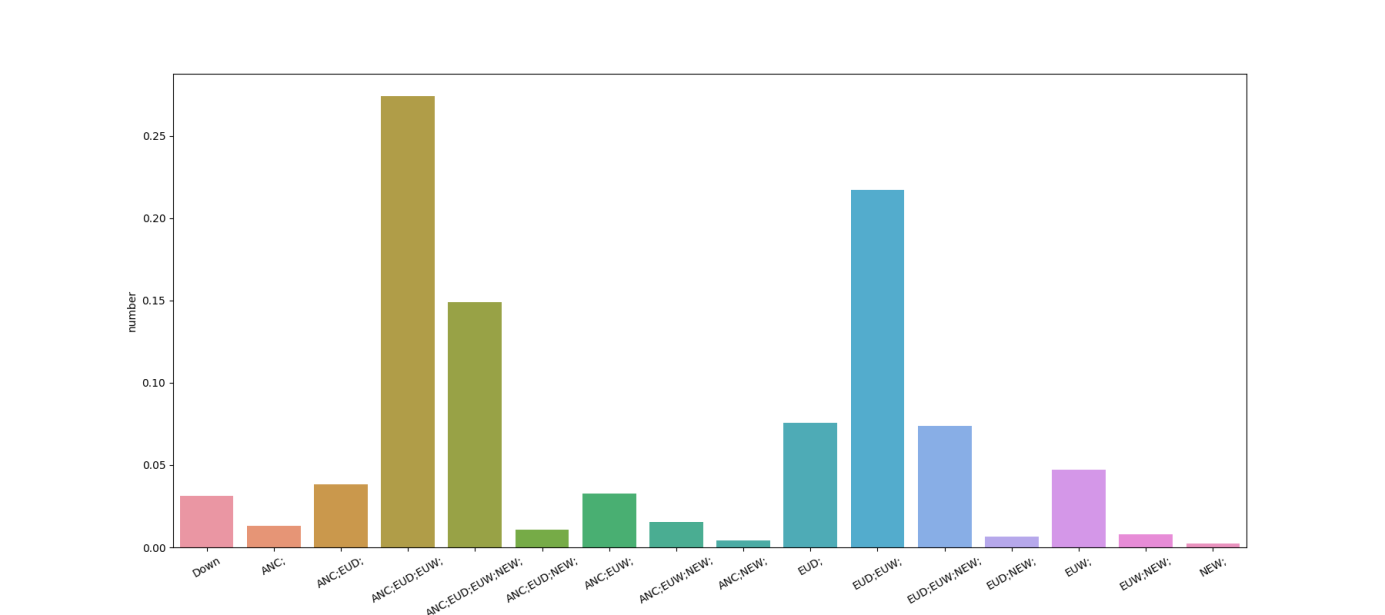


A

B

C

Figure 9. Reference bias towards geographical/status groups in the reference panel for the genotypes imputed by Beagle5 that were not present in the HQ genotypes (IMP1). (A) KD033, (B) KD037, (C) VEM185.


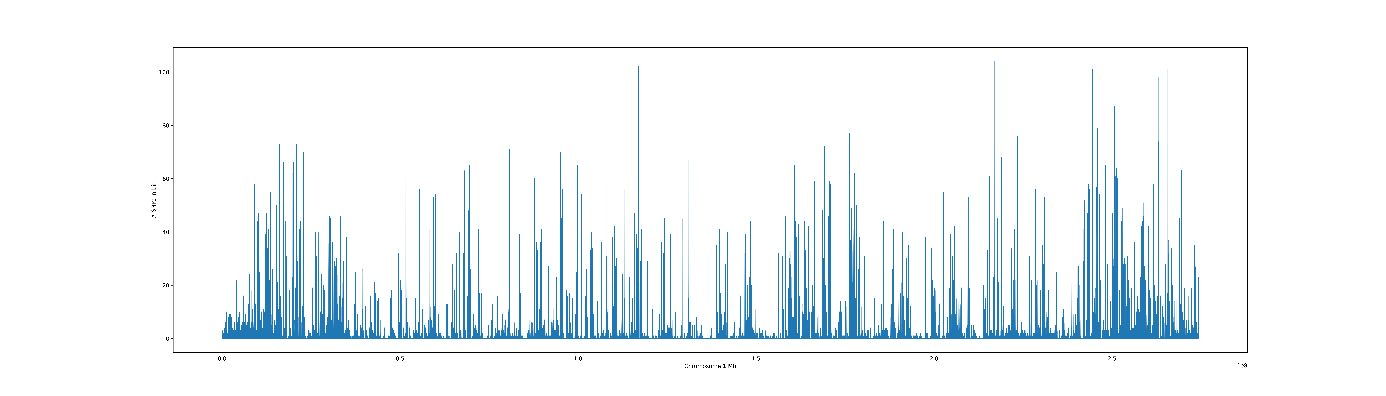


Figure 10. The amount of incorrectly imputed genotypes (not occuring in HQ) throughout the autosomes.

# Appendix ROH


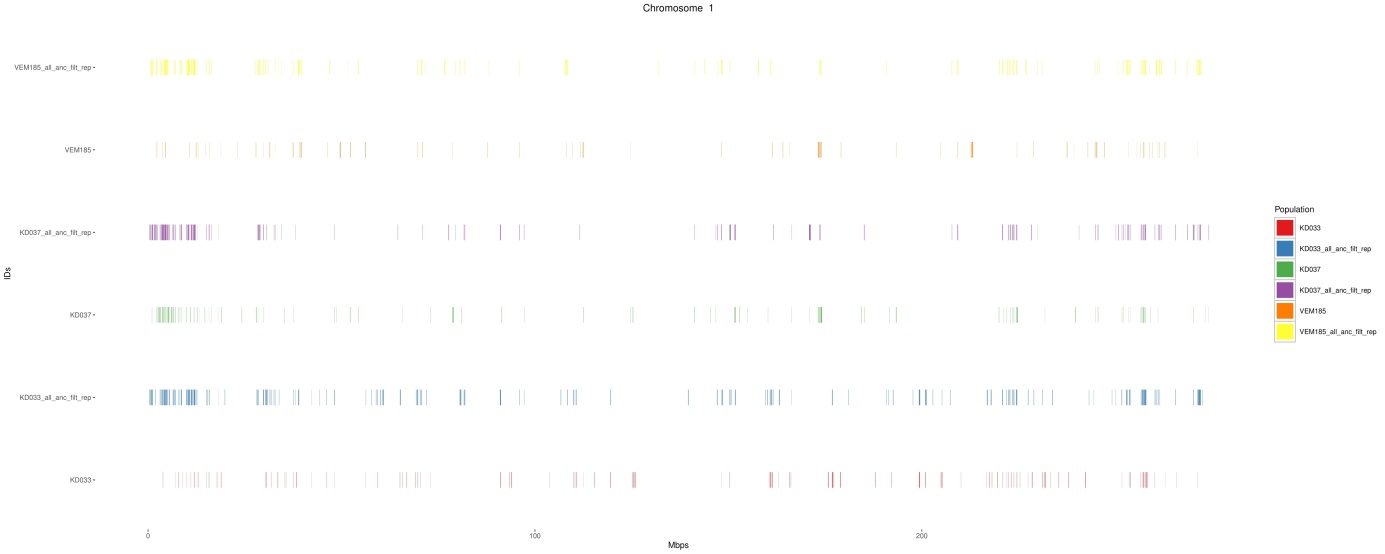

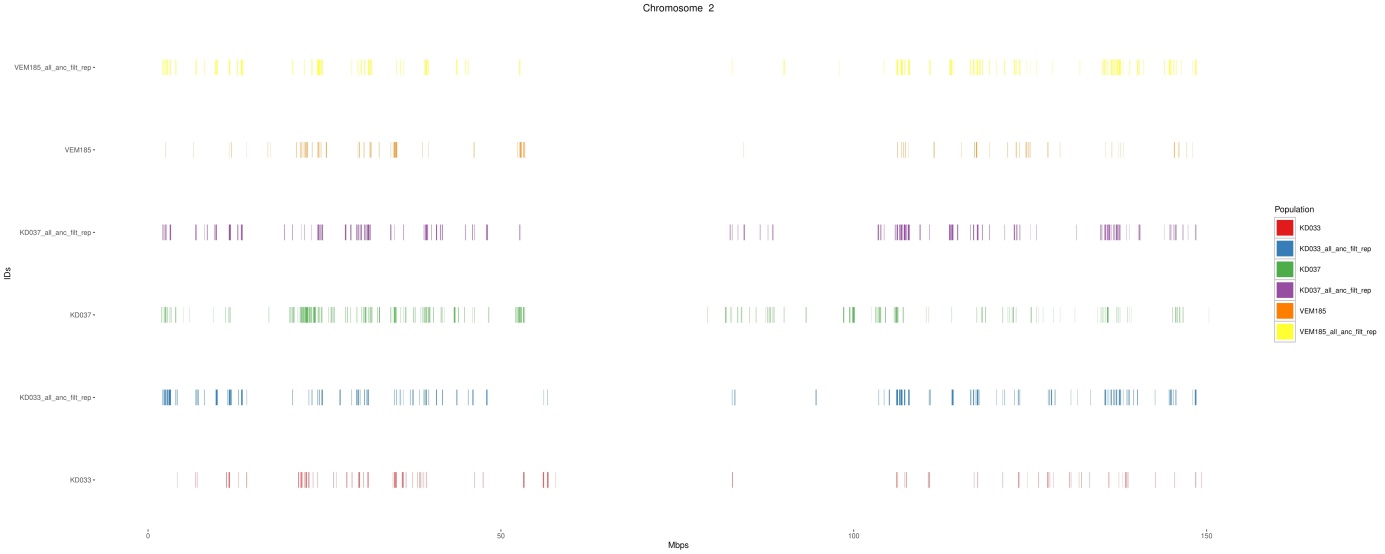

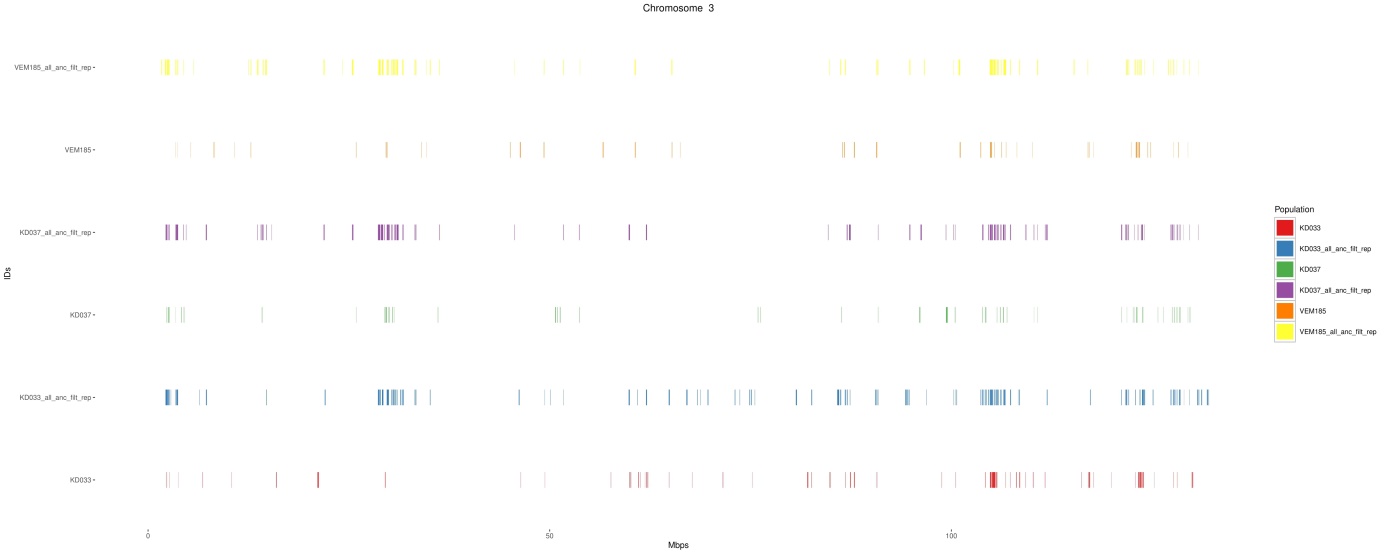

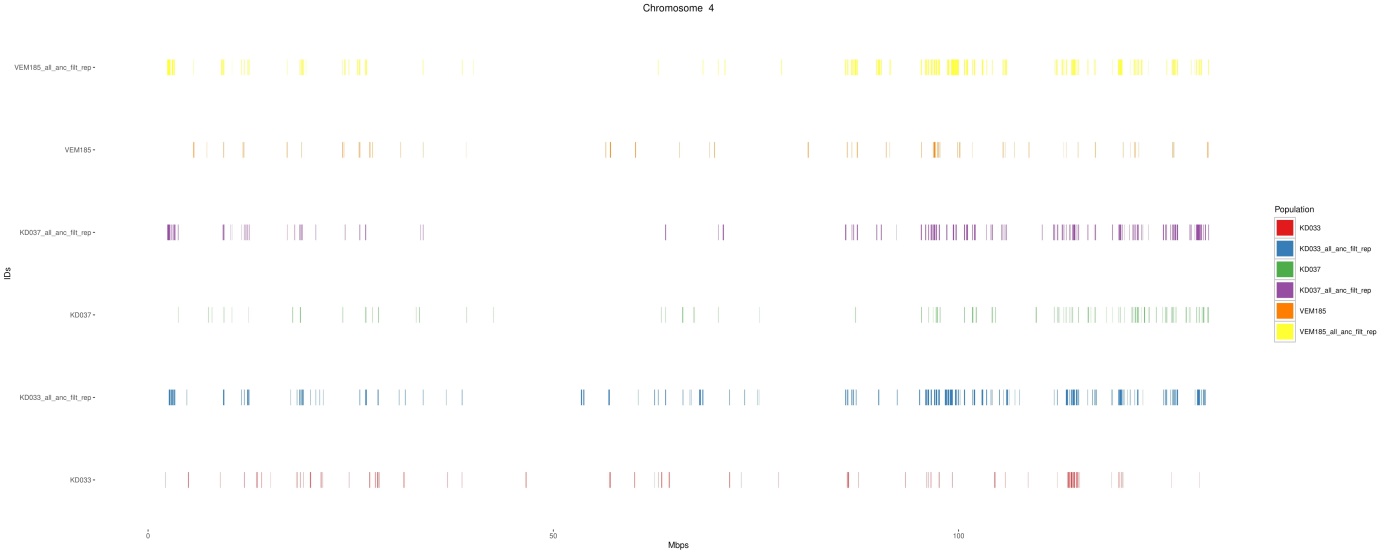

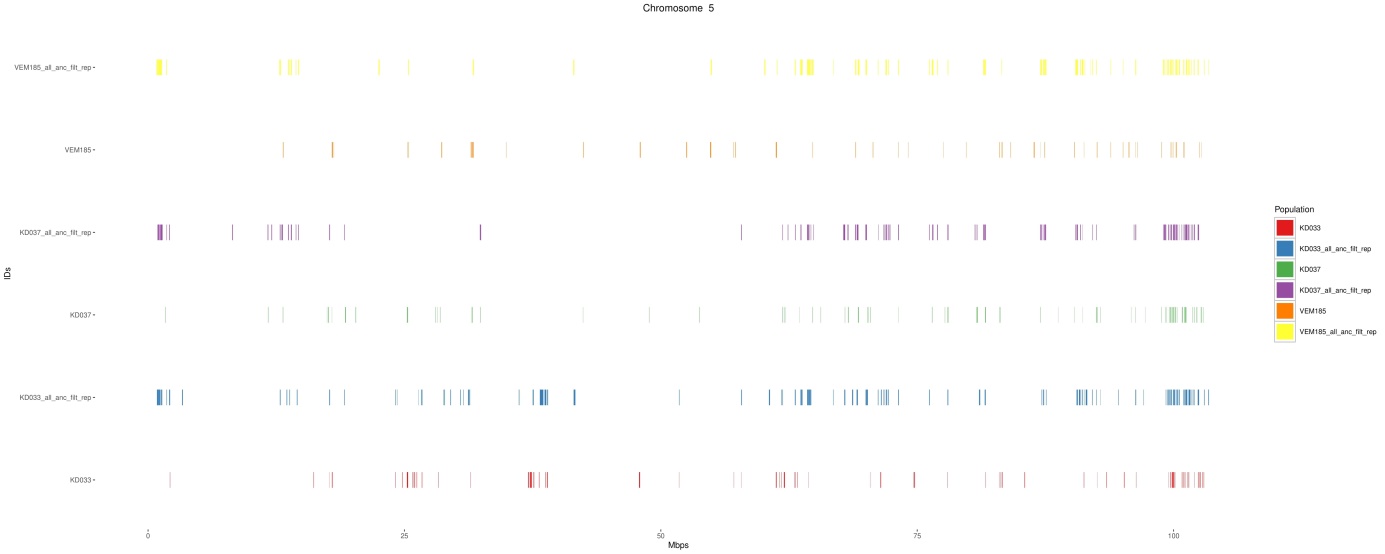

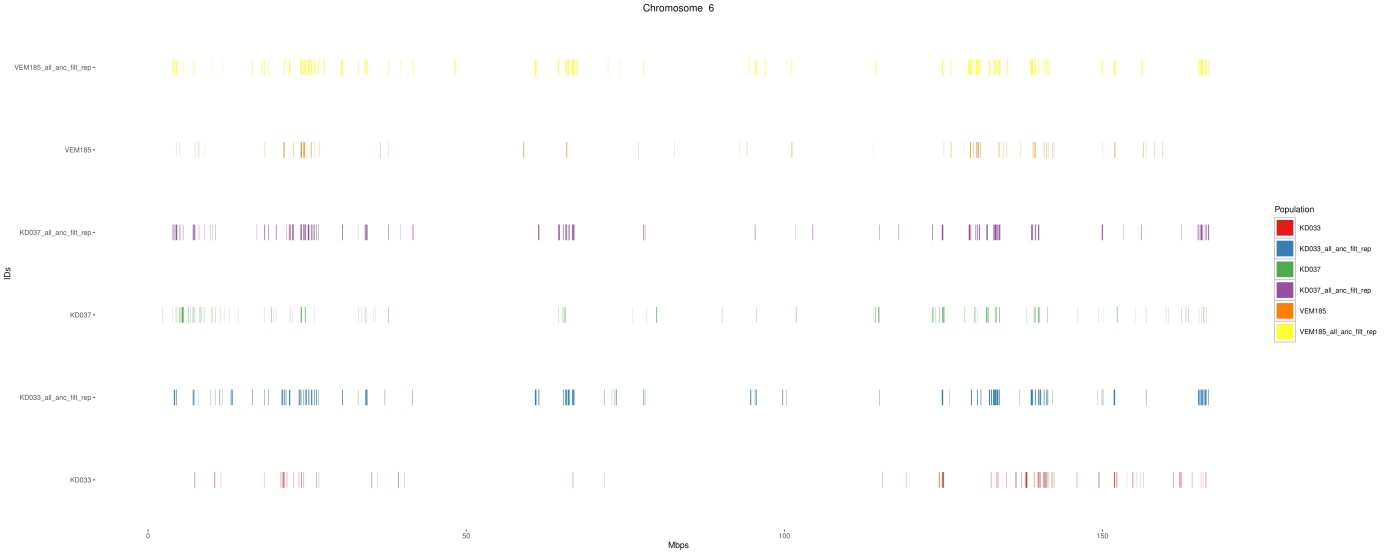

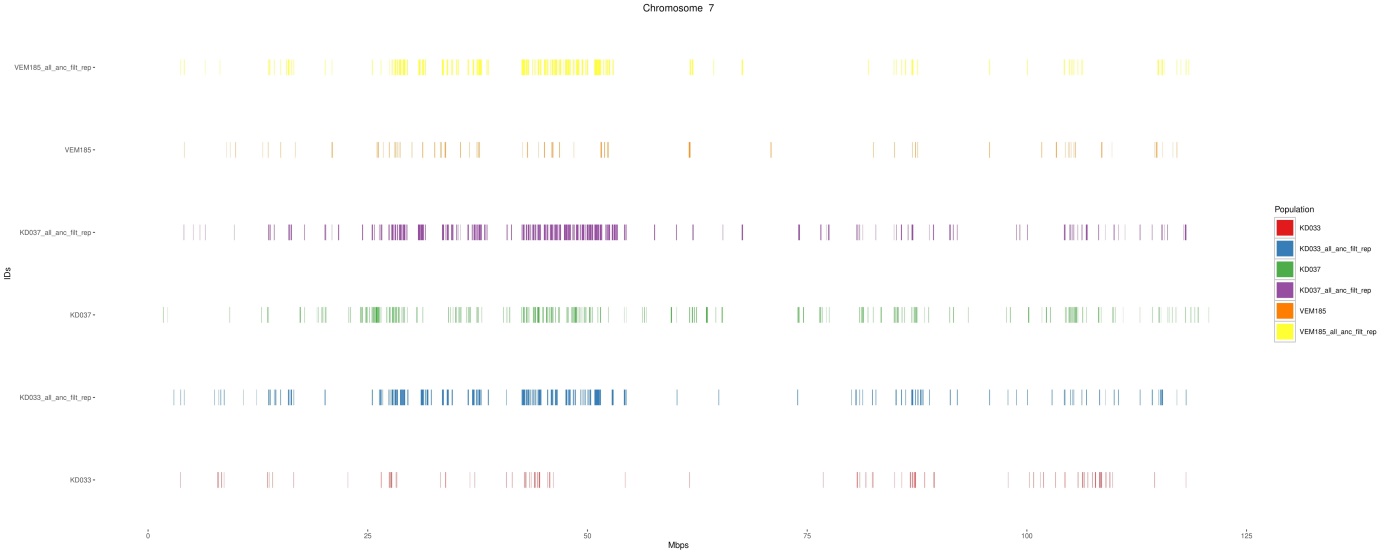

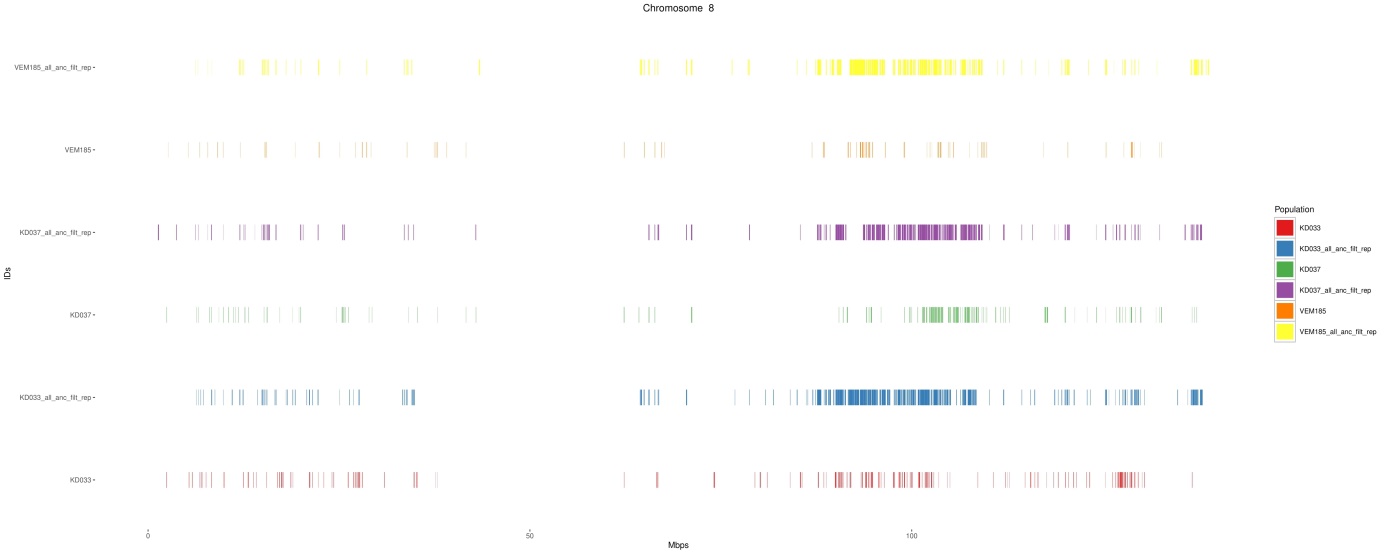

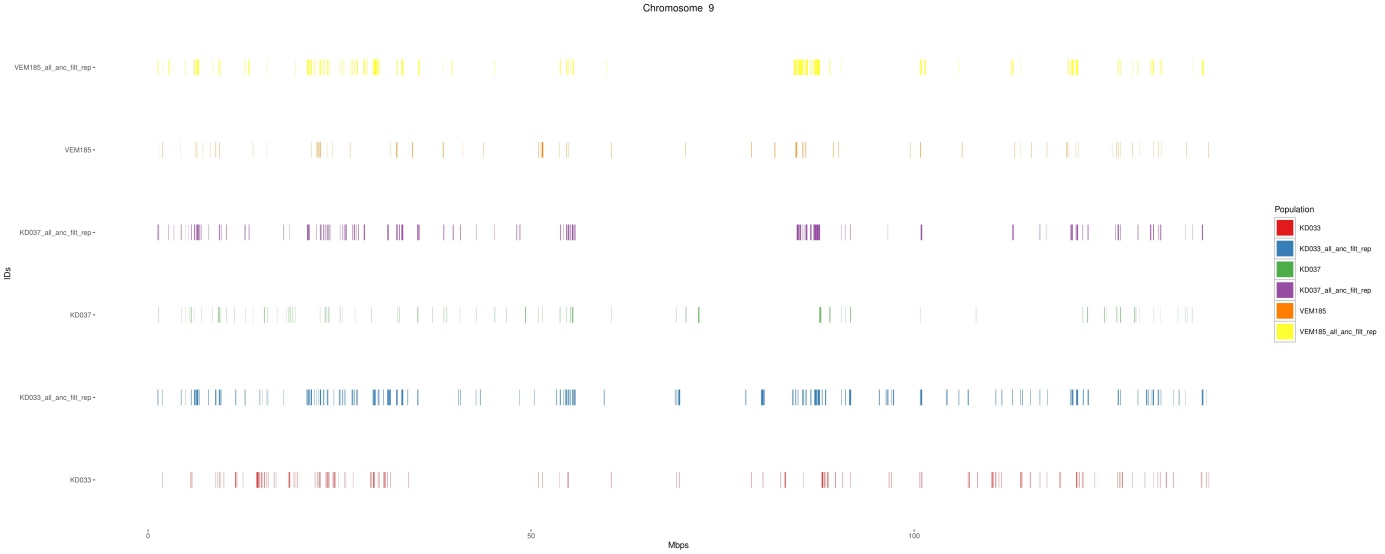

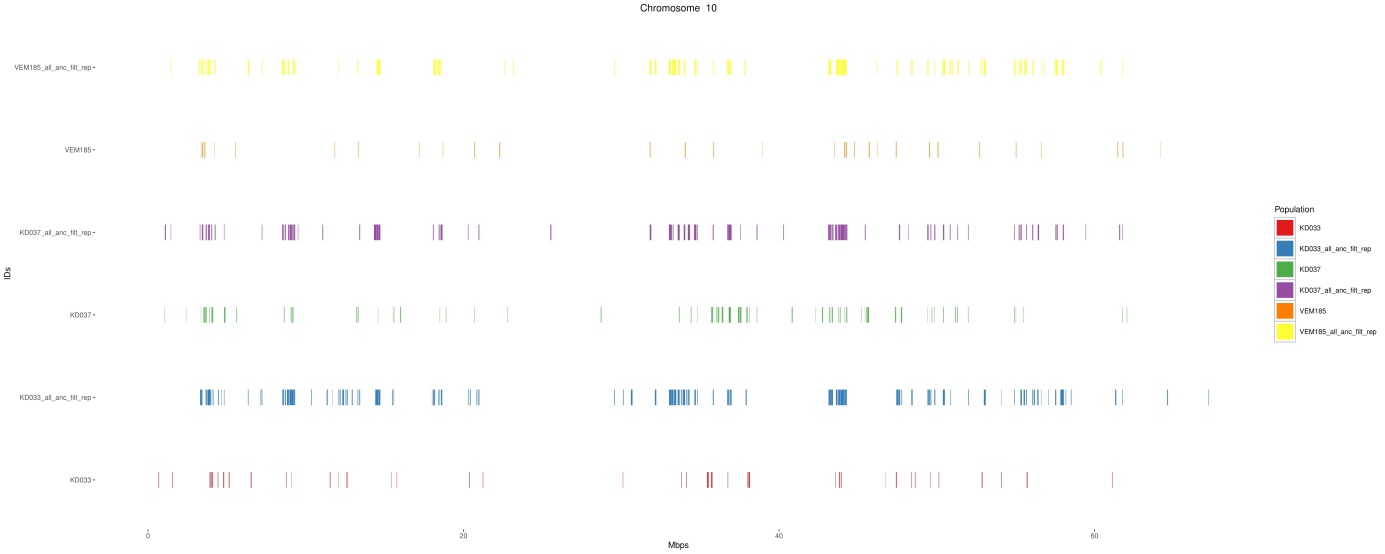

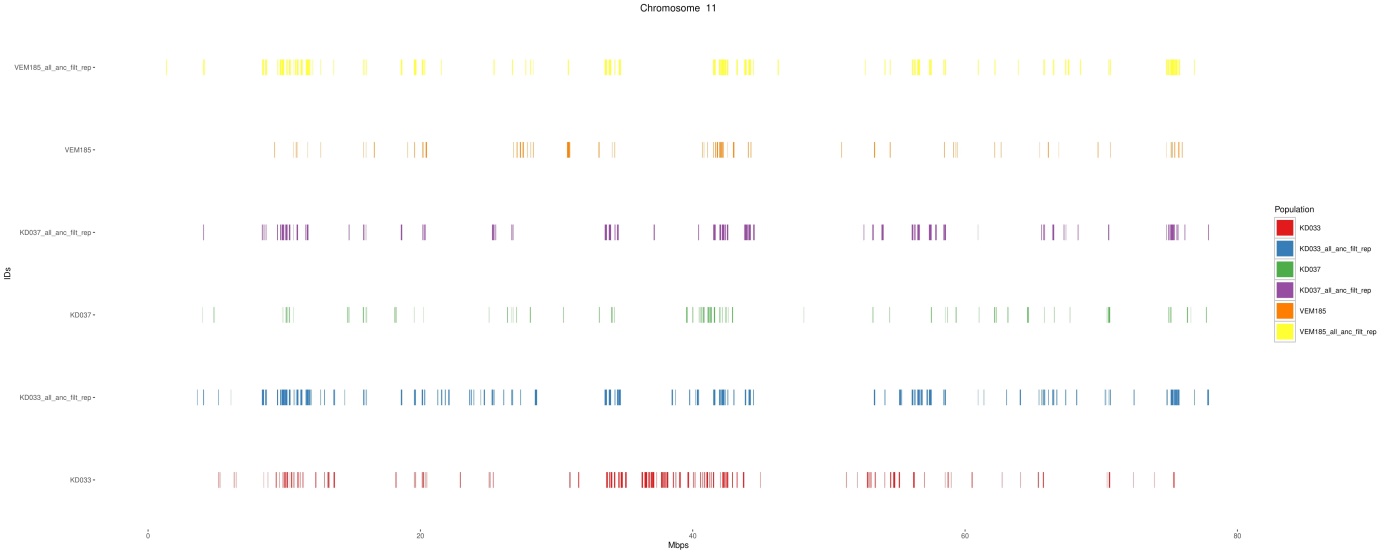

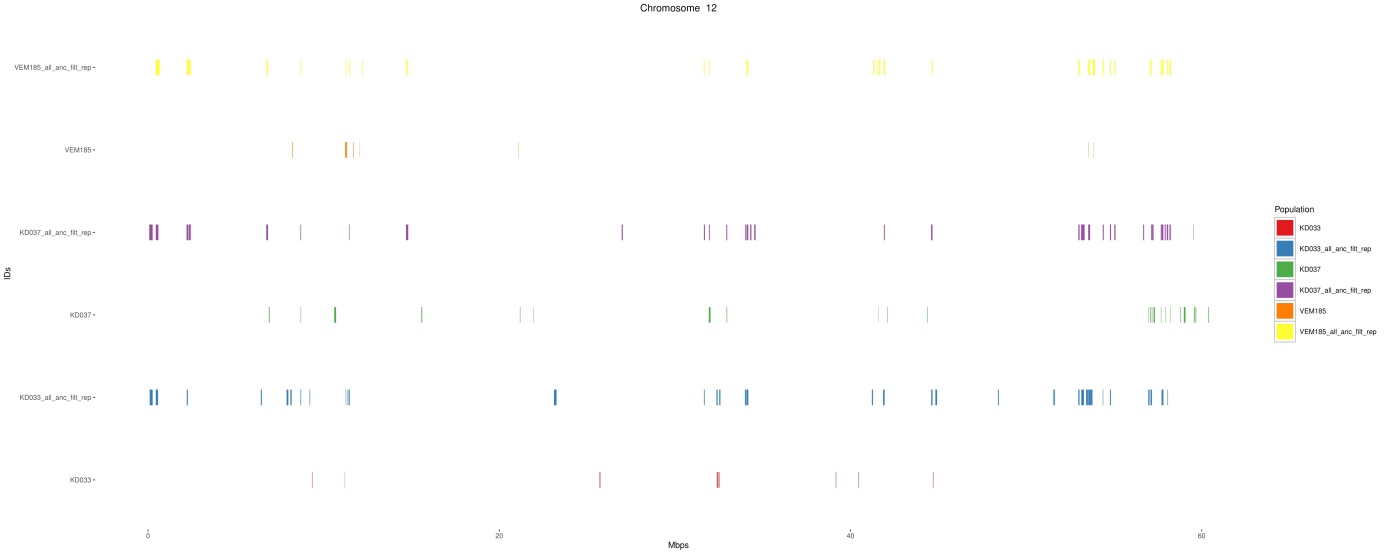

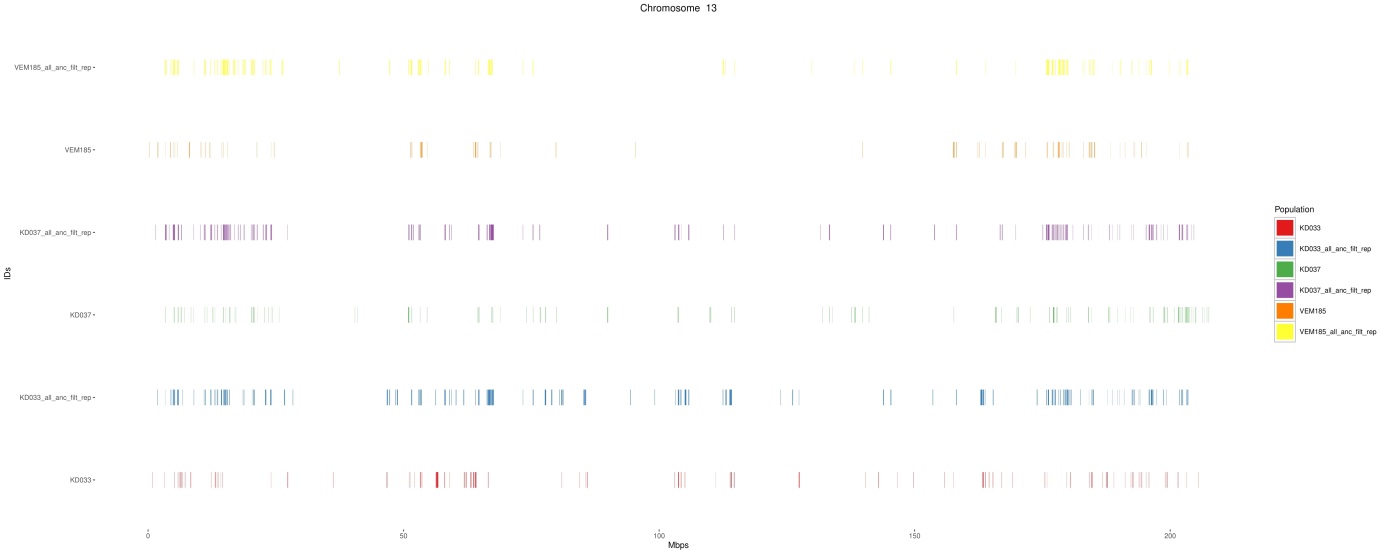

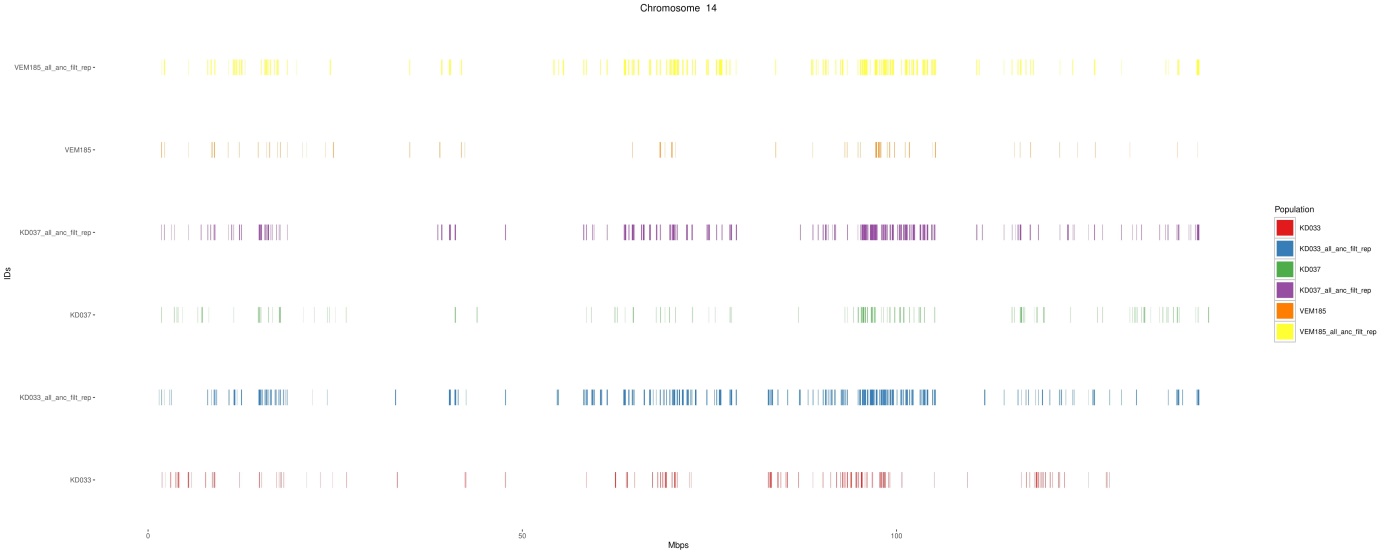

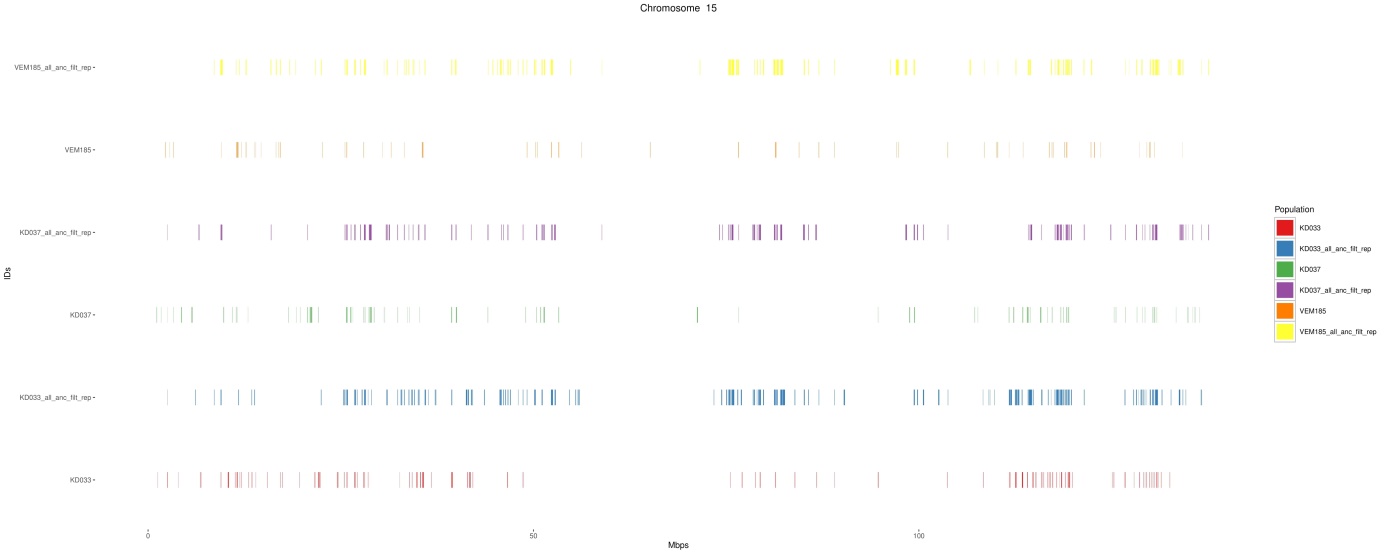

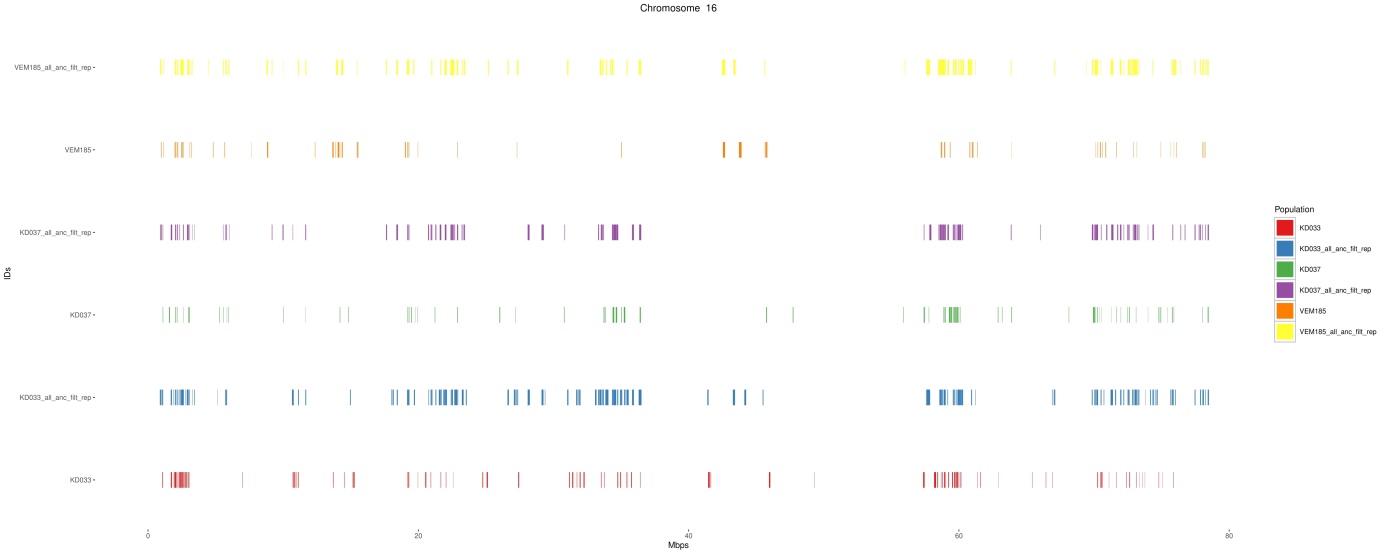

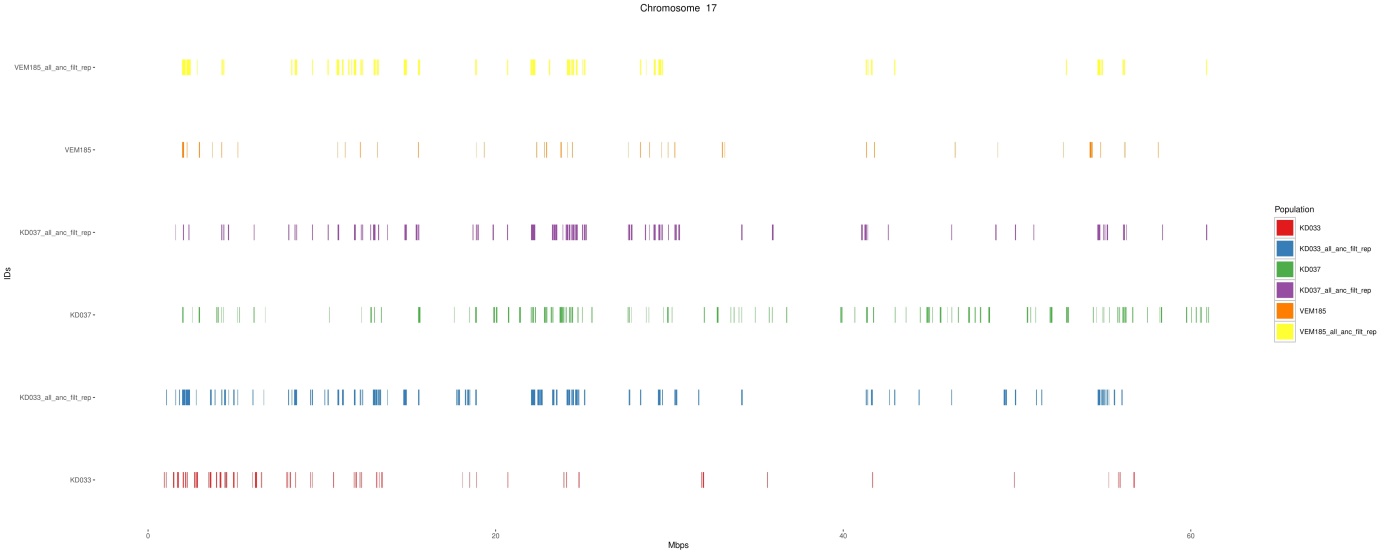

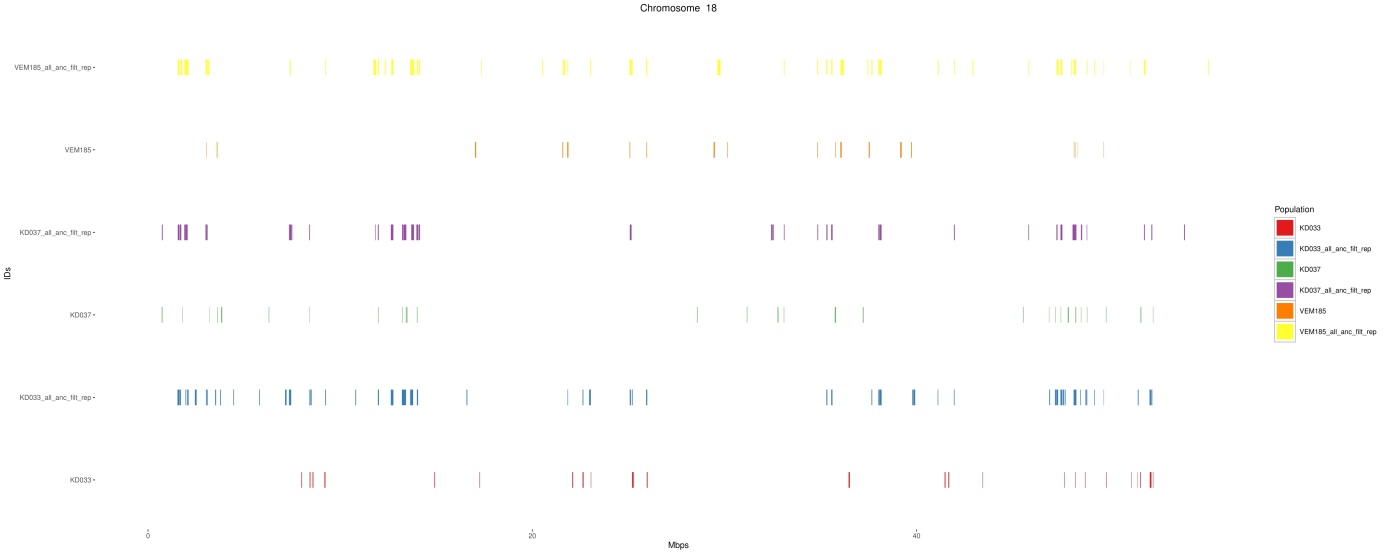


Figure 1. ROH bins throughout the chromosome for chromosome 1-18. All anc filt rep refers to the imputed samples using the IMP1 scheme
